# An Electroactive Oligo-EDOT Platform for Neural Tissue Engineering

**DOI:** 10.1002/adfm.202003710

**Published:** 2020-08-14

**Authors:** Kaja I. Ritzau-Reid, Christopher D. Spicer, Amy Gelmi, Christopher L. Grigsby, James F. Ponder, Victoria Bemmer, Adam Creamer, Ramon Vilar, Andrea Serio, Molly M. Stevens

**Affiliations:** Department of Materials, Department of Bioengineering, Institute of Biomedical Engineering, Imperial College London, London SW7 2AZ, UK; Department of Materials, Department of Bioengineering, Institute of Biomedical Engineering, Imperial College London, London SW7 2AZ, UK; Department of Medical Biochemistry and Biophysics, Karolinska Institutet, Stockholm 171 77, Sweden; Department of Chemistry, York Biomedical Research Institute, University of York, Heslington YO10 5DD, UK; Department of Materials, Department of Bioengineering, Institute of Biomedical Engineering, Imperial College London, London SW7 2AZ, UK; Applied Chemistry and Environmental Science, School of Science, RMIT University, Melbourne 3000, Australia; Department of Medical Biochemistry and Biophysics, Karolinska Institutet, Stockholm 171 77, Sweden; Department of Chemistry, Imperial College London, London SW7 2AZ, UK; Department of Materials, Department of Bioengineering, Institute of Biomedical Engineering, Imperial College London, London SW7 2AZ, UK; Department of Materials, Department of Bioengineering, Institute of Biomedical Engineering, Imperial College London, London SW7 2AZ, UK; Department of Chemistry, Imperial College London, London SW7 2AZ, UK; Department of Materials, Department of Bioengineering, Institute of Biomedical Engineering, Imperial College London, London SW7 2AZ, UK; Centre for Craniofacial & Regenerative Biology, King’s College London and The Francis Crick Institute, Tissue Engineering and Biophotonics Division, Dental Institute, King’s College London, London SE1 9RT, UK; Department of Materials, Department of Bioengineering, Institute of Biomedical Engineering, Imperial College London, London SW7 2AZ, UK; Department of Medical Biochemistry and Biophysics, Karolinska Institutet, Stockholm 171 77, Sweden

**Keywords:** 3,4-ethylenedioxythiophene, biomaterials, electrospinning, neurite outgrowth, tissue engineering

## Abstract

The unique electrochemical properties of the conductive polymer poly(3,4-ethylenedioxythiophene):polystyrene sulfonate (PEDOT:PSS) make it an attractive material for use in neural tissue engineering applications. However, inadequate mechanical properties, and difficulties in processing and lack of biodegradability have hindered progress in this field. Here, the functionality of PEDOT:PSS for neural tissue engineering is improved by incorporating 3,4-ethylenedioxythiophene (EDOT) oligomers, synthesized using a novel end-capping strategy, into block co-polymers. By exploiting end-functionalized oligoEDOT constructs as macroinitiators for the polymerization of poly(caprolactone), a block co-polymer is produced that is electroactive, processable, and bio-compatible. By combining these properties, electroactive fibrous mats are produced for neuronal culture via solution electrospinning and melt electrospinning writing. Importantly, it is also shown that neurite length and branching of neural stem cells can be enhanced on the materials under electrical stimulation, demonstrating the promise of these scaffolds for neural tissue engineering.

## 1 Introduction

To investigate fundamental questions in human brain development and disease, tissue engineering approaches have employed 3D polymer based biomaterial scaffolds as guidance cues to grow 3D neuronal cultures in conditions resembling the in vivo environment.^[1–5]^ Yet, fully recapitulating all functional aspects of neuronal networks within an in vitro system still presents significant challenges, due to the complex physiological environment of the brain. An ideal biomaterial for complex functional neural cultures must be able to provide both precise architectural control to guide neural growth and effective electrical communication to interface with electrically excitable neural cells.^[6]^ There is therefore a pressing need for new 3D electroactive biomaterials able to mediate neural tissue engineering.

Advances in bio-fabrication technologies have led to promising developments in specialized 3D architectures for neural tissue engineering, including 3D scaffolds with microscale or nanoscale topography to guide cellular interactions.^[7–11]^ Techniques such as electrospinning have been used to create biomimetic fibrous matrices which have successfully been applied for the growth of neural networks, as suitably aligned nanoscale fibers can provide directionality for the growth of neurons.^[9,12–17]^ More recently, melt electrospinning writing (MEW) has emerged as a promising method to deposit highly defined fibrous scaffolds.^[18]^ This technique has so far been minimally used in neural tissue engineering, yet has numerous advantages including reproducible and precise control over fiber diameter and scaffold architecture. MEW therefore provides exciting opportunities for specific cell guidance and the creation of networks with precise alignment and directionality.^[19]^ The absence of solvents during fabrication by MEW also minimizes toxicity issues during cell culture.

Incorporating electroactive functionality into neural scaffolds has been shown to aid recapitulation of in vivo biological environments.^[20,21]^ Neurons are connected to each other via synapses, which are used to translate changes in membrane potential into biochemical messages that are exchanged between neighboring neurons and propagate ongoing signals within the network. Although the membrane potential is determined by ion exchange, exogenous electrical stimulation can be a useful tool to interface with living neurons, and in previous studies it has been shown to enhance neuronal differentiation and neurite outgrowth.^[20,22,23]^ Conjugated polymer (CP)-based materials have therefore recently gained significant attention in the field of neural tissue engineering due to their capacity to conduct charge through the transport of both electrons and ions.^[24–26]^ The versatility and flexibility of polymer synthesis also enables the production of varied scaffold architectures, in contrast to traditional metal electrodes, bridging the communication gap between biology and electronics.^[27–31]^ When compared to metal electrodes, CPs have been shown to be beneficial for neural tissue engineering due to their ability to modulate material stiffness and impedance to better match that of neural tissue.^[32]^ The lower stiffness of CPs compared to metal electrodes has been shown to improve long-term contact with neuronal cells and the electrode, by reducing the mechanical mismatch and reactive tissue response to stiff metal devices, prolonging electrical stimulation and extending beneficial effect on neuronal growth.^[24,32,33]^ This can potentially be further improved by incorporating CPs into conjugated hydrogel (CH) materials, as hydrated hydrogel networks provide an ideal substrate for 3D cell culture, recreating the environment of native soft tissue, and allowing the diffusion of nutrients and signaling biomacromolecules.^[34–36]^ Electroactive hydrogels have been shown to permit electrical stimulation of cells in 3D and to mediate biological signaling.^[27,37–39]^ However, continued issues with low conductivity have hindered translation of CHs into biomedical applications.^[24,40,41]^

In the last decade, poly(3,4-ethylenedioxythiophene):polystyrene sulfonate (PEDOT:PSS) has dominated the CP field, due to its excellent electrical properties, chemical and thermal stability and low oxidation potential.^[24,25]^ However, despite the promise of CPs as attractive materials for in vitro models of functional brain tissue, challenges with their use continue to hinder widespread application in tissue engineering. Difficulties in functionalization, lack of biodegradability, poor material reproducibility due to low solubility and processability, and inadequate mechanical properties are particularly prominent.^[25,42]^

Recently, electroactive oligomers have emerged as a promising alternative to full length CPs.^[42,43]^ Typically they share similar electrical properties after doping to the corresponding conducting polymer formed from doping of the parent CP, yet have more potential to install versatile chemical functionality for further derivatization.^[44,45]^ The use of conjugated oligomers has several desirable advantages. First, they provide a precisely defined structure that is in stark contrast to the polydisperse nature of CPs. This provides improved control and homogeneity of material structure and function, which is essential to elucidate the underlying mechanisms of signal transduction at the material-cell interface. Second, the greatly improved solubility and processability offered by oligomeric structures provides opportunities to exploit many emerging techniques for advanced biomaterial preparation, and for synergistically regulating physical, topographical, and electrical cues within a single scaffold.^[45,46]^ Finally, unlike full length CPs, short oligomers, typically <10 monomers, can be consumed by macrophages, allowing the production of materials that are both electroactive and biodegradable. The benefits of degradable scaffolds for both in vivo and in vitro applications have been highlighted by a number of recent reports demonstrating the benefits of material remodeling and degradation by growing cells on tissue development, as well as for the long term tolerance of hybrid materials.^[45,47–49,52,53]^

In 2002, Schmidt and colleagues first demonstrated the feasibility of incorporating pyrrole oligomers into a fully biodegradable polymer, via degradable ester linkages.^[47]^ Recent studies from this group reported an oligoaniline based electroactive polymer, with simple and scalable synthesis and purification, that could be used for electrochemically triggered delivery of anti-inflammatory drugs.^[50,51]^ Aniline oligomers have also been explored for use in electroactive biodegradable scaffolds for soft tissue repair, including a dopant-free conjugated elastomer polymer, generated by chemically linking conjugated aniline oligomers, biodegradable poly(caprolactone) (PCL) segments and a dopant component.^[52]^ Polyester-based scaffolds are particularly attractive due to the tunability of polymer degradation rate based on monomer composition.^[36,53]^ However, it has so far proven difficult to apply such an approach to PEDOT due to the reported chemical instability of oligoEDOTs and the lack of free functional groups for further functionalization. Our group have recently offered a potential solution to this problem, by reporting the synthesis of precisely defined, glyoxyl-capped, bifunctional oligoEDOT constructs that are stable, possess tunable properties, and provide diverse reactive handles for further derivatization.^[54]^

In the present study, we utilized our oligoEDOT constructs to provide a modular platform for biomaterial synthesis. End functionalized oligoEDOTs were used as macroinitiators for the synthesis of PCL block co-polymers and as crosslinkers for hydrogelation. We show that solvent electrospinning and MEW can be used to fabricate fibrous scaffolds, with defined nanotopography. This represents a major advantage of our approach, as these methods cannot typically be applied directly to PEDOT-based materials, due to their poor processability. Moreover, we demonstrate that oligoEDOT-PCL is a permissive substrate for neuronal cell culture, and that electrical stimulation enhances neurite length and branching of neuronal cells.

## 2 Results and Discussion

### 2.1 Synthesis of End-Functionalized OligoEDOTs

**Figure 1:**
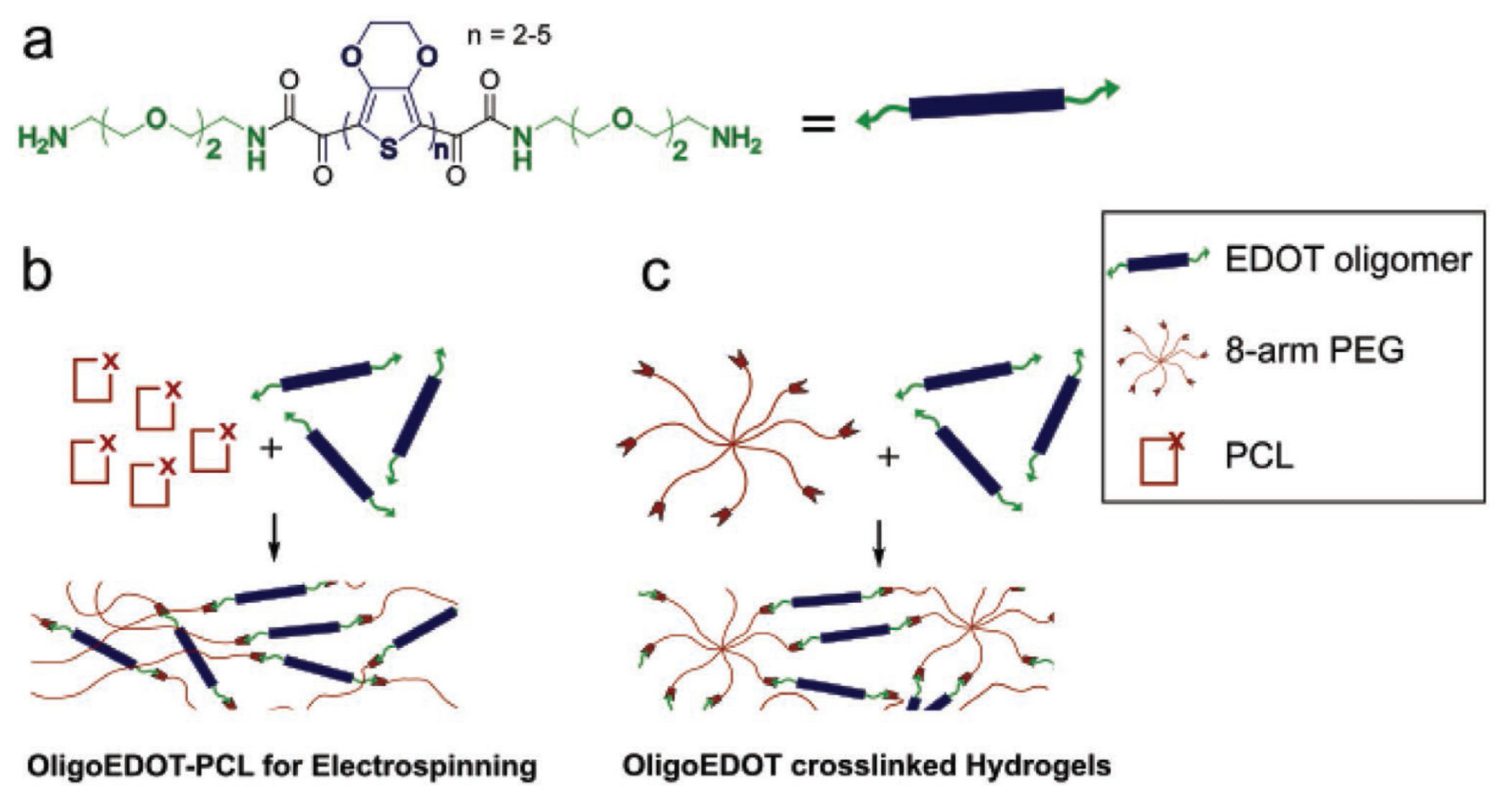
Schematic use of oligoEDOTs as components of functional biomaterials. a) Amine-capped oligoEDOTs synthesized during this study, *n* = 2–5. b) Use of amino-oligoEDOT as an initiator for ring-opening polymerization to generate fibrous ABA-block co-polymers with poly(caprolactone) (PCL). c) Crosslinking of multi-arm polyethylene glycol (PEG) macromers to form oligoEDOT based hydrogels.

To construct end-functionalized oligoEDOTs we adopted a synthetic approach recently reported by our group.^[54]^ The iterative process consists of thiophene glyoxylation, bromination, chain extension, and oligomer cross-coupling, and yields end-functionalized EDOT oligomers of defined chain lengths (Figure S1, Supporting Information). We previously demonstrated that a variety of functional end-capping handles could be easily incorporated, enabling the generation of a diverse range of functionalized alkoxy-thiophene monomers and oligomers.^[54]^ We reasoned that amino derivatives would provide a versatile reactive handle for further derivatization, while incorporation of a short triethylene-glycol linker would provide flexibility and enhanced solubility, facilitating processing and subsequent modification (Figure 1a). After treatment of EDOT with oxalyl chloride, the intermediate glyoxylyl chloride **1**, was reacted with a mono-Boc (tert-butyloxycarbonyl) protected, diaminotriethylene-glycol linker **2**, to generate the *N*-protected, mono-functionalized EDOT monomer **3** (Figure S1, Supporting Information). Subsequent bromination yielded di-functional monomer **4**, which could undergo subsequent chain extension and oligomer couplings via palladium-catalyzed direct arylation (Figure S1, Supporting Information). The use of direct arylation, over alternative strategies such as Stille or Kumada couplings, limited potential problems with poor functional group compatibility and residual catalyst toxicity.^[55]^ Through this strategy, we were able to generate a range of protected amine-functionalized oligoEDOTs in an iterative manner, with defined chain lengths (*n* = 2–5, **5**–**8**) (Figure S1, Supporting Information).

### 2.2 oligoedot-pcl characterization

**Figure 2:**
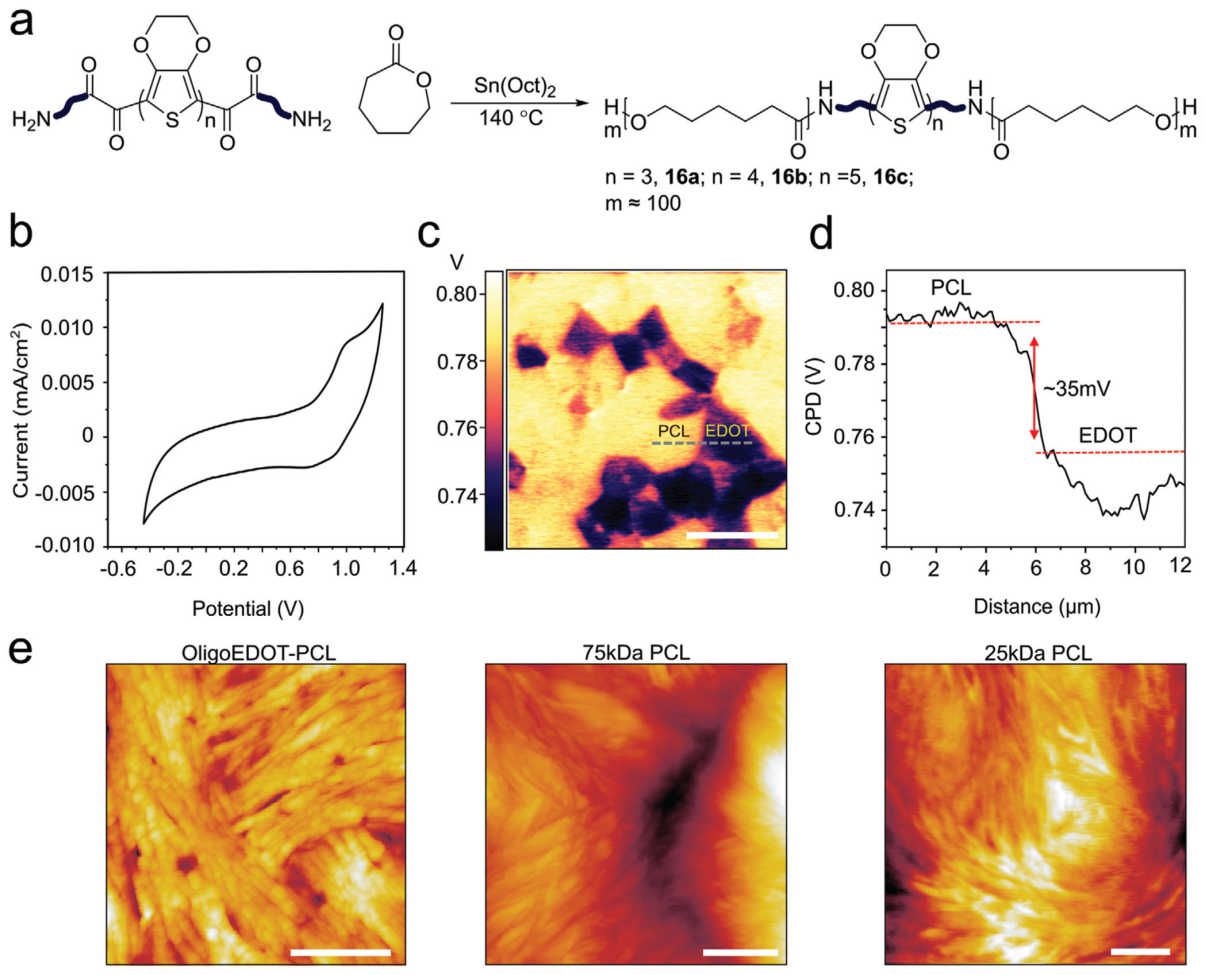
OligoEDOT-PCL polymerization and characterization. a) oligoEDOT-PCL synthesis by ring opening polymerization. b) Cyclic voltammogram (100 mV s^−1^) of tetramer oligoEDOT-PCL in propylene carbonate with 0.5 M tetrabutylammonium hexafluorophosphate as the supporting electrolyte. c) Kelvin probe force microscopy (KPFM) image of tetramer oligoEDOT-PCL spin coated film. Scale bar: 10 μm d) KPFM line scan of surface potential along the black dashed line. e) Tapping mode AFM image of tetramer oligoEDOT-PCL, high *M*_w_ PCL and low *M*_w_ PCL. Scale bars: 500 nm.

To prepare constructs suitable for further processing and scaffold fabrication, we selected PCL as a suitable co-polymer, due to its biodegradable properties and well-established use in tissue engineering. Boc-protected dimer (**5**), tetramer (**7**), and pentamer (**8**) oligoEDOTs were deprotected under acidic conditions to yield the free amines (**9**, **11**, and **12** respectively), (Figure S1, Supporting Information). Ring-opening polymerization of *ε*-caprolactone was then undertaken using the amino-oligoEDOT as a macroinitiator (Figures 1b and 2a). The new ABA block co-polymer structures (subsequently named oligoEDOT-PCL **16**a–c) were synthesized with a total molecular weight of ≈25 kDa (i.e., two PCL chains of ≈12 kDa off a central oligoEDOT core of ≈1kDa, weight dictated by monomer:oligoEDOT ratio). This molecular weight was chosen to provide sufficient PCL for improved processability while maximizing the electroactivity of the oligoEDOT block. To investigate the optoelectronic properties of the oligoEDOT-PCL constructs, UV–vis spectra were recorded on thin films (Figure S2, Supporting Information). Chemically synthesized PEDOT in the neutral (undoped) state typically absorbs in the visible region from 400 to 600 nm.^[56]^ Accordingly, the maximum absorbance in samples of the three oligoEDOT-PCL films were between 450 and 500 nm, with the absorbance spectra red-shifted with increasing oligomer chain length as expected with increasing conjugation length (Figure S2, Supporting Information). The optical band gap (*E*_g,opt_) was calculated from the onset of absorption for the three oligoEDOT-PCL films, which ranged from 2.48 eV for diEDOT-PCL to 2.03 eV for pentaEDOT-PCL, as further detailed in Table S1, Supporting Information. These observations are consistent with the optical spectra range of the parent amino-oligoEDOTs and the structures recently reported by us.^[54]^ This confirmed that incorporation of the oligoEDOT into a PCL ABA block co-polymer did not significantly alter the optical properties of the EDOT-block.

Next, we conducted cyclic voltammetry to elucidate the electrochemical properties of dimer, tetramer and pentamer oligoEDOT-PCL films in organic and aqueous solutions (Figure 2b and Figure S3, Supporting Information). TetraEDOT-PCL **16** films provided the most stable electrochemical behavior over several samples. We therefore used tetraEDOT-PCL for more in-depth thin film characterization, scaffold preparation, and neuronal cell culture. The tetraEDOT-PCL film exhibited a broad anodic (0.8–1.1 V) and a broad cathodic (0.4–1 V) peak, with tetrabutylammonium hexafluorophosphate (TBAPF_6_) as the supporting electrolyte at a scan rate of 0.1 V s^−1^, demonstrating redox activity corresponding to the formation of a radical cation (Figure 2b).

To further examine the electronic properties of the oligoEDOT-PCL films, we applied Kelvin probe force microscopy (KPFM) to investigate changes in surface potential at the localized micro-scale. Such experiments provide a more accurate reflection of the local electrical environment experienced by individual cells than bulk material measurements. Figure 2c shows a heterogenous surface potential map of the tetraEDOT-PCL **16**b polymer film, with a local surface potential, *V*_CPD_, obtained from the contact potential difference (CPD) between the conductive tip and sample. Distinct regions of lower and higher work functions were evident on the surface of the film. Figure 2d illustrates a line scan along the black dotted line, depicting the difference in work function between the bright and dark areas. These results suggest nanoscale aggregation of the conjugated oligoEDOT on the PCL co-polymer surface.

Subsequently, we used atomic force microscopy (AFM) scanning to further examine the effects of polymerizing PCL with conjugated EDOT oligomers on surface topography (Figure 2e). TetraEDOT-PCL films were compared with pure PCL films with a comparable molecular weight ≈25 kDa, and high molecular weight PCL ≈c75 kDa. High molecular weight PCL films displayed lamellar structures, which aggregated into compact spherules of ≈1.5–2 m in size, consistent with previous reports (Figure 2e).^[57]^ Image scans of low molecular weight PCL films showed a similar surface morphology, with evidence of more striated lamellar, indicating increased crystallinity, consistent with the reduced molecular weight. AFM scans of tetraEDOT-PCL revealed distinct differences in the surface morphology compared to both pure PCL samples, as individual fibrils of ≈40 nm were resolved on the polymer surface. These image scans demonstrate that the inclusion of EDOT oligomers influences the surface morphology. Additionally, by blending oligoEDOT-PCL films with higher molecular weight PCL at different blend ratios, we were able to tune the morphological properties of the films (Figure S4, Supporting Information). Polymer blending has emerged as a versatile technique to adjust material properties, and has previously been used to combine certain favorable material properties.^[58]^ By blending oligoEDOT-PCL with high molecular weight PCL, we demonstrate the possibility to adjust the mechanical properties of the material according to the application and scaffold type. It is important to note that the addition of increasing volumes of insulating PCL will also negatively influence the electrical properties of the material. Further studies are required to elucidate the PCL:oligoEDOT ratio window in which both beneficial electroactivity and enhanced material processability are maintained.

### 2.3 OligoEDOT Crosslinked Hydrogels

We next wanted to test whether our oligoEDOTs could be effectively used as hydrogel crosslinkers, to demonstrate the versatility of our approach for materials preparation. We therefore set out to undertake the gelation of complementary polyethylene glycol (PEG) macromers (Figure 1c). While amine salts **9**–**12** were able to provide partial water solubility, upon buffering to physiological pHs the oligomers quickly became insoluble at the concentrations required for hydrogelation. Attempts to form hydrogels with 8-arm PEG succinimide esters therefore yielded highly heterogeneous, mechanically weak gels encapsulating areas of precipitated oligomer (Figure S5, Supporting Information). We therefore sought to make use of the synthetic versatility of our amino-oligoEDOT series to introduce water solubilizing functionalities.

Thiol groups provide convenient reactive handles for hydrogelation via a number of mechanisms, including nucleophilic Michael addition and radical thiol-ene reactions.^[58]^ These reactions often proceed with high specificity and rapid reaction kinetics, leading to their widespread use in the biomaterial community. Moreover, it has been widely shown that simple changes in gelation conditions such as pH and temperature, or alterations to thiol or alkene chemistry, can dramatically influence gel properties providing tunability.^[60–62]^ Amino-triEDOT **10** was therefore derivatized with cysteine **13** in an attempt to both enhance water solubility and increase hydrogelation efficiency (Figure S6, Supporting Information). We attempted gelation with 8-arm PEG-maleimide, however water solubility continued to limit efficiency and gel homogeneity. A Glu-Glu-Cys tripeptide **14** was therefore ligated at the oligomer termini to provide additional charge at physiological pH (Figure S6, Supporting Information). Functionalized oligomer **15** was found to be water soluble over a wide pH range, even at the high concentrations necessary for hydrogelation. Mechanically stable hydrogels (5% by polymer weight) were immediately formed upon mixing with 8-arm PEG-maleimide in PBS (Figure S5, Supporting Information). A slight improvement in gel homogeneity could be achieved by undertaking gelation at pH 6, due to an increase in gelation time that allowed efficient mixing. Gel stability was high, as monitored through leaching of the triEDOT crosslinker **15** out of the gel (Figure S7, Supporting Information). Although a slight pH dependence on gel swelling ratio (relative to the lyophilized gels) was observed, the effect was minimal and likely due to the presence of the EEC tripeptide (Figure S8, Supporting Information).

The same peptide-modified oligomer strategy was then applied to the formation of tetra- and penta-EDOT crosslinked gels. However, the increase in oligomer length was found to be sufficient to reduce water solubility and prevent gelation, with oligomer precipitation resulting instead. For this reason, further hydrogel processing and characterization was not pursued, and we instead chose to focus on the generation of fibrous oligoEDOT-based scaffolds as described in subsequent sections.

These results highlight the importance of overcoming the hydrophobicity of extended *π*-systems to allow the synthesis of electroactive hydrogels.^[27]^ Although this hydrophobicity IS limiting in the work presented here, the high modularity of our oligoEDOT derivatization strategy offers a potential means to address this difficulty. As demonstrated above, water solubilizing chains can be easily ligated to oligomer building blocks. Furthermore, we have previously demonstrated that our oligoEDOT synthesis strategy is applicable to alternative dialkoxythiophene monomers.^[54]^ The use of reported water soluble EDOT derivatives, bearing carboxyl or sulfonate group is particularly attractive.^[63–65]^ It is therefore likely that optimization of this process will allow the future synthesis of tetra- and penta-oligomers with sufficient solubility for gelation.

### 2.4 OligoEDOT-PCL Films Support Neural Stem Cell Growth and Differentiation

**Figure 3:**
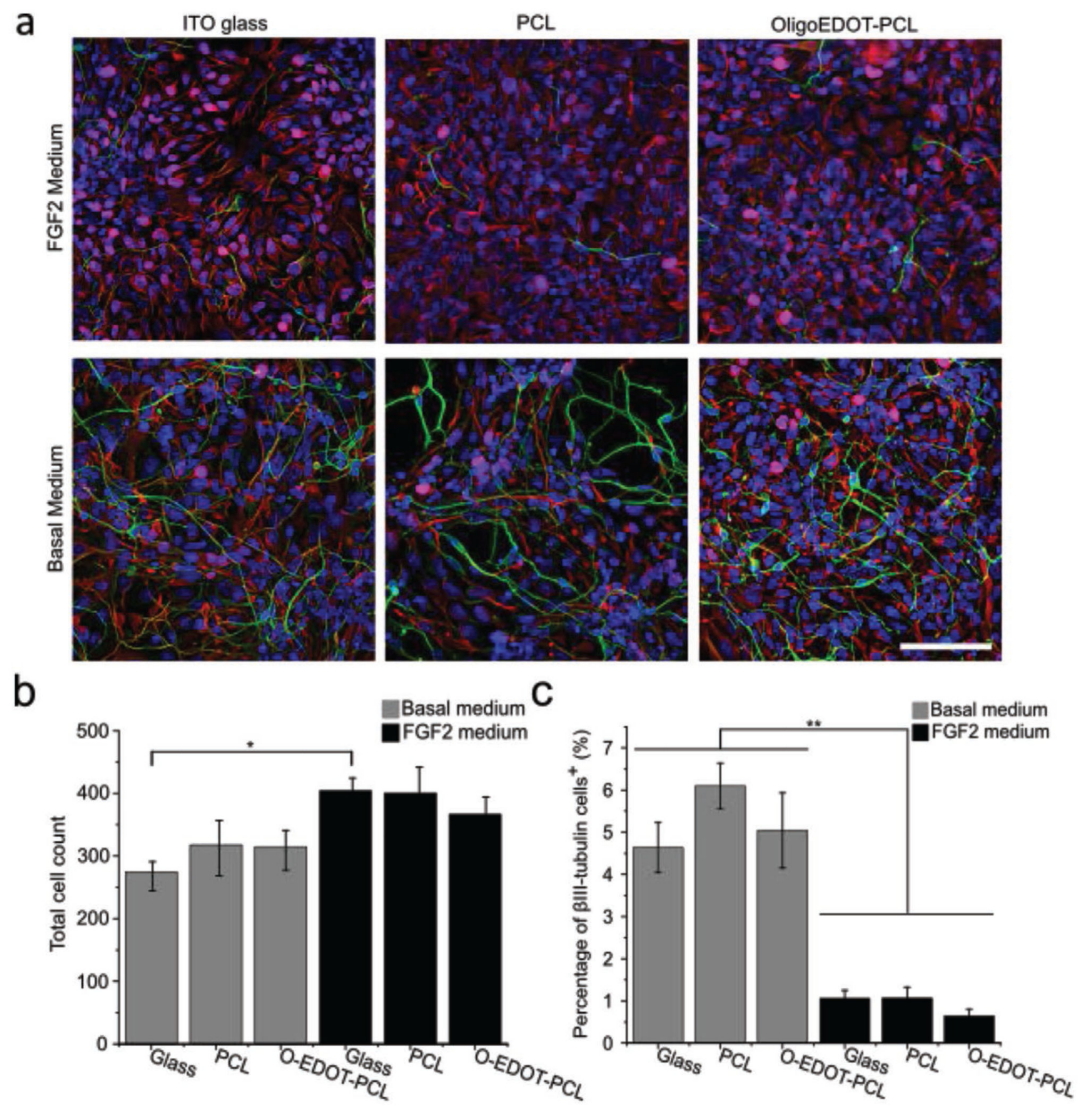
OligoEDOT-PCL scaffolds are biocompatible and support NSC proliferation and differentiation. a) Widefield fluorescence images of NSCs cultured on oligoEDOT-PCL, PCL, and ITO glass at day 7 either in basal media (to promote differentiation) or FGF2 media (to promote proliferation) (*β*III-tubulin, green; nestin, red; Ki67, magenta; DAPI, blue). Scale bar: 100 μm. b) Total cell count of NSCs on substrates after 7 days. c) Neuronal differentiation on substrates as evaluated by the percentage of *β*III-tubulin^+^ cells over the total number of cells after 7 days. (Data shown as mean ∓ S.E.M. *N* = 2, *n* = 3 with a minimum of 15 images analyzed for each condition. One-way ANOVA with post hoc Tukey’s test was used. * represents *p* < 0.05 and ** represents *p* < 0.001).

To investigate the suitability of oligoEDOT-PCL films as substrates for neural scaffolds, human iPSC-derived neural stem cells (NSCs) were seeded onto spin-cast films.^[66]^ Seeded scaffolds were cultured in fibroblast growth factor-2 (FGF2) containing media to promote NSC proliferation, or media without any mitogens to promote differentiation (Basal Medium) (Figure 3a).^[67]^ After 7 days of culture, cells were stained for *β*III-tubulin (a marker of differentiated neurons), nestin (a marker of neuronal progenitors and stem cells) and ki-67 (a proliferation marker). The addition of FGF2 media showed that cells were proliferating and promoted the expression of ki-67 (Figure 3a). The total cell count on the oligoEDOT-PCL films in basal and FGF2 media showed no significant difference to the PCL scaffolds or glass controls (Figure 3b). Immunostaining revealed a significant increase in *β*III-tubulin positive cells cultured in basal media on all substrates, with no significant difference being observed between NSCs cultured on oligoEDOT-PCL films compared to the control groups (Figure 3c). Together, these results demonstrate that oligoEDOT-PCL films are biocompatible and are a suitable substrate for NSC differentiation and proliferation.

### 2.5 Scaffold Preparation of OligoEDOT-PCL

**Figure 4:**
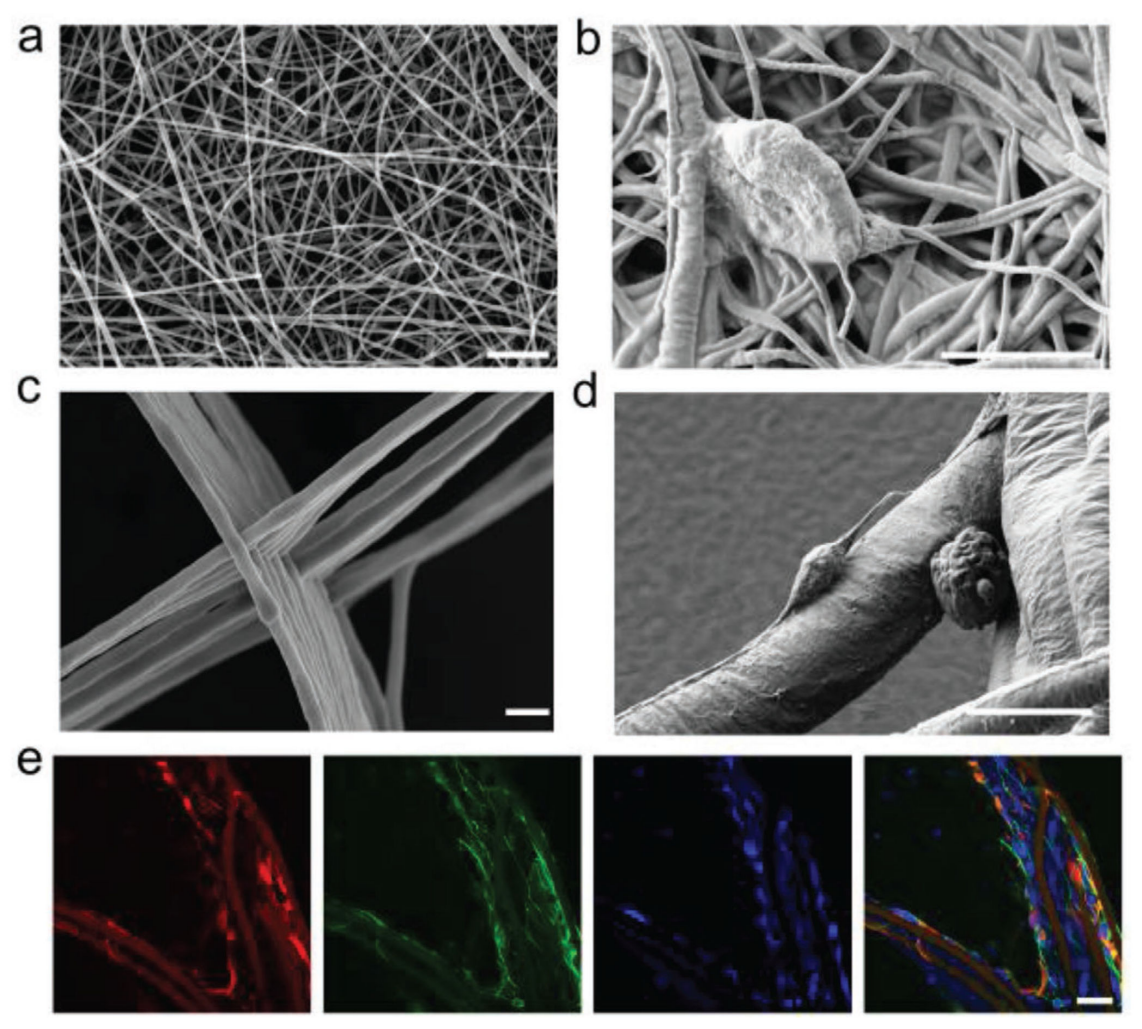
3D oligoEDOT-PCL scaffold fabrication. a) SEM image of oligoEDOT-PCL scaffold created using solution electrospinning. Scale bar: 20 m. b) NSC differentiation on solution electrospun scaffold after 24 h. Scale bar: 5 μm. c) Scanning electron microscopy (SEM) image of oligoEDOT-PCL lattice scaffold created using melt electrospinning writing (MEW). Scale bar: 20 μm. d) Neural stem cell (NSC) differentiation on melt electrospun 3D lattice scaffold after 24 h. Scale bar: 10 μm. e) Confocal laser scanning microscopy image of NSCs on melt electrospun 3D lattice scaffold for 24 h stained with *β*III-tubulin (green), nestin (red) and DAPI (blue). Scale bar: 20 μm.

Electrospun fibrous membranes have been used extensively in tissue engineering applications, due to the architectural resemblance of micrometer diameter fibers to the fibrils found in native extracellular matrix.^[68]^ To generate electroactive 3D fibrous scaffolds from our oligoEDOT-PCL constructs, membranes were prepared using solvent electrospinning, and MEW. Typically, these methods cannot be applied directly to PEDOT due to its poor solubility and processability. Our block co-polymer approach therefore offers an opportunity to address these limitations and construct electroactive, fibrous, EDOT-based scaffolds for neuronal culture. For solution electrospinning, tetraEDOT-PCL films were blended at a 50% ratio with high molecular weight PCL, and polymer solutions were spun at a 20% w/v concentration in a 9:1 mixture of CHCl_3_: CH_3_OH, following our previously reported protocol.^[58]^ This resulted in the deposition of pink colored fibrous mats after electrospinning (Figure S9, Supporting Information). Fiber diameters were measured by scanning electron microscopy (SEM), with an average diameter of ≈400 nm (Figure 4a). In order to assess the suitability of the produced mats for neuronal culture, NSCs were seeded on electrospun tetraEDOT-PCL scaffolds and cultured in basal medium to promote differentiation for 24 h prior to fixing (Figure 4b). Immunostaining for *β*III-tubulin and nestin indicated that NSCs adhered to the membrane and underwent neuronal differentiation (Figure S10, Supporting Information). Interestingly, the oligoEDOT-PCL fibers were found to be fluorescent in the red channel (Figure S10, Supporting Information). We reasoned that this may be due to the shear forces or exposure to high voltages during electrospinning, affecting the molecular properties of the polymer. Current studies in our group are trying to elucidate the physical processes that occur to cause this change in oligomer emission.

Recent studies have reported that low pore size in electrospun scaffolds can act as a barrier to cell penetration.^[69,70]^ Studying cell behavior in complex 3D environments can also be challenging due to randomly orientated fibers and heterogenous architectures.^[70]^ To investigate the suitability of oligoEDOT-PCL for the production of scaffolds with defined microarchitectures, we also used MEW to construct a 5 cm × 5 cm 3D lattice, which comprised of 10 stacked layers, with an approximate spacing of 100 μm between grids and a fiber diameter of 5 μm (Figure 4c). Interestingly, the scaffold color here was gold, further suggesting that factors during the fabrication process, such as shear stress and voltage, may have an effect on the molecular packing of the oligoEDOT-PCL polymer (Figure S11, Supporting Information).

We examined cell adherence by seeding NSCs on oligoEDOT-PCL scaffolds for 24 h in differentiation media, and SEM imaging revealed that NSCs adhered to individual fibers in the scaffold (Figure 4d). Consistent with previous studies, neurites within our scaffolds preferentially extended in the same directions as the fibers (Figure 4d,e).^[9,13–15,17]^

### 2.6 Electrical Stimulation Enhances Neurite Outgrowth

**Figure 5:**
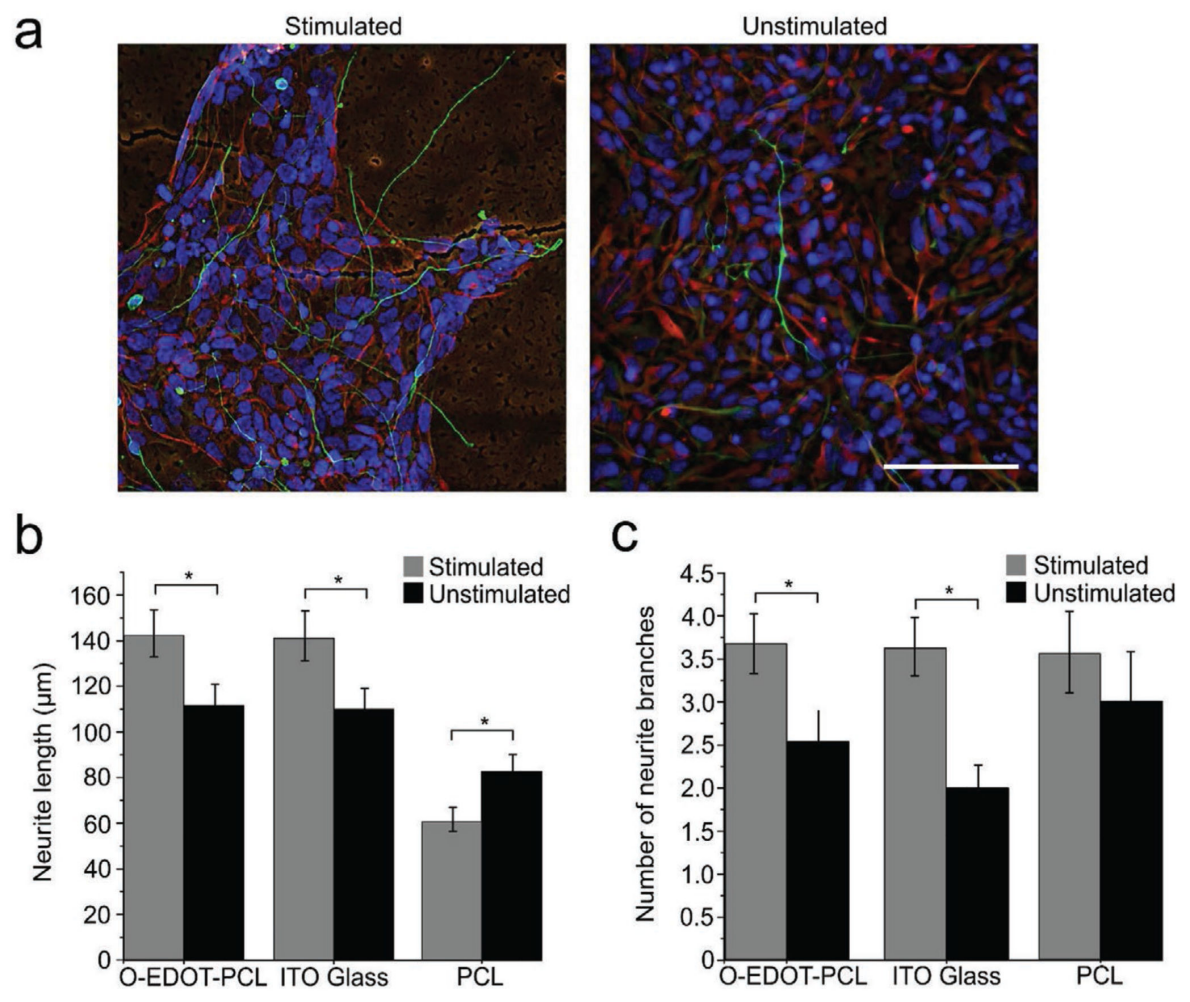
Effect of electrical stimulation (ES) on NSCs cultured on oligoEDOT-PCL films. a) Widefield fluorescence images of stimulated (left panel) and unstimulated (right panel) (*β*III-tubulin, green; nestin, red; DAPI, blue). Scale bar: 100 μm. b) Mean neurite length of NSCs on substrates with or without electrical stimulation. c) Neurite branching of NSCs on substrates with or without electrical stimulation. (Data shown as mean ∓ S.E.M. *N* = 3, *n* = 2 with a minimum of 12 images analyzed for each condition. Student’s *t*-test was used. * represents p < 0.05).

The key advantage of CP-based scaffolds for neural tissue engineering are the opportunities they provide to electrically stimulate cells. Previous studies have shown that switching the redox state of CPs by applying a voltage can change neuronal behavior, including cell adherence, proliferation, and differentiation, though the precise mechanism is not yet fully understood.^[20,71,72]^ However, the poor mechanical properties of conventional CPs can limit the ability to fabricate complex scaffolds and sustain long term electrical stability. Our oligoEDOT platform provides an exciting opportunity to overcome this challenge. To investigate the effects of electrical stimulation on NSCs cultured on oligoEDOT-PCL films, we examined neurite length and branching in NSCs after applying a pulsed direct current (DC) (Figure 5a). To prepare the films, tetraEDOT-PCL **16b** films were deposited by spin coating directly on to indium tin oxide (ITO) glass, a conductive substrate serving as the working electrode. We chose to use PCL films spin coated on ITO glass as the non-conductive control to eliminate possible contributions from electrochemical processes in the cell culture medium. For the conductive control, bare ITO was used, which has previously been used as a substrate to stimulate neural cells in vitro and has been shown to evoke an electrical response in cultured neurons.^[73]^ An electrode cell assembly, constructed in-house, was used for in vitro NSC stimulation (Figure S12, Supporting Information), and NSCs were seeded in the chambers for 24 h prior to stimulation. A platinum wire counter electrode was placed inside the cell culture medium in the micro chambers, at a distance of 1 cm from the oligoEDOT-PCL films. To better mimic physiological conditions of neural network activity, we used pulsed electrical stimulation. We also reasoned that this would avoid a build-up of charge, thereby allowing long term electrical stimulation, limiting any adverse effects on cell viability. Trains of 1 ms pulses of 600 mV at 1 Hz were applied for 24 h, followed by fixing and immunostaining of cells.

We observed an increase in mean NSC neurite length following stimulation on tetraEDOT-PCL films (142.1 ± 10.4 μm) compared to unstimulated tetraEDOT-PCL films (111.4 ± 8.7 μm) (Figure 5b and Figure S13, Supporting Information). Similarly, neurite length increased when NSCs were stimulated on ITO glass control substrates, compared to the unstimulated group (140.1 ± 10.7 and 109.9 ± 8.06 μm respectively) (Figure 5b and Figure S13, Supporting Information). Surprisingly, neurite length was found to decrease upon stimulation of NSCs on the PCL negative control group (60.5 ± 5.2 and 82.4 ± 6.45 μm) for stimulated and unstimulated groups respectively, emphasizing the important role of the oligoEDOT block in promoting neurite extension. Neurite branching was similarly increased on stimulated oligoEDOT-PCL films and ITO control substrates, compared to their respective unstimulated groups (Figure 5c).

These results are consistent with previous studies which have reported an increase in neurite outgrowth following electrical stimulation on conventional CPs.^[20,74–77]^ Our substrates, exhibiting greatly improved processability and more versatile material properties, therefore offer an exciting alternative to pure CP scaffolds for stimulating neuronal cultures. It has previously been proposed that cellular changes in response to electrical stimulation are initiated at the cell surface, altering cell surface receptors and protein adsorption, or modulating the growth cone morphology.^[78,79]^ In our study, it is also plausible that electrical stimulation causes changes in the redox states of the EDOT oligomer, changing the bulk properties and surface tension of the polymer, thereby causing changes in NSC behavior. Further investigations into the origins of these effects are currently underway. Critically, such studies are facilitated by the precisely defined molecular structure provided by our oligoEDOT strategy.

## 3 Conclusion

In a search for electroactive materials suitable for the fabrication of complex architectures in tissue engineering, we have developed a new electroactive ABA block co-polymer, oligoEDOT-PCL. We have achieved a route to synergistically apply electrical cues and create controlled topographies by combining the electroactive properties of oligoEDOT structures, with the favorable processability of PCL. The combination of these features is critical to achieving more complex architectures for in vitro models of the developing brain, and we are currently exploring the application of this material to develop highly defined scaffolds to guide 3D neuronal growth. Future work should focus on harnessing the well-defined molecular structure offered by our oligoEDOT synthesis strategy for precise control over chemical functionalization, and the redox active properties for controlled delivery of soluble growth factors and charged small molecules. This will provide the opportunity to better mimic native tissue and provide spatio-temporally controlled chemical guidance cues, such as patterning factors. Significantly, this study demonstrates that oligoEDOT based biomaterials have potential for neural tissue engineering, by providing a modular platform for biomaterial synthesis, thereby improving processability and the potential to generate complex 3D architectures for tissue engineering scaffolds.

## 4 Experimental Section

Details of oligoEDOT synthesis and their PCL co-polymers, electrical stimulation device design and setup are included in the Supporting Information.

*Preparation of OligoEDOT-PCL FILMS*: Thin films were prepared by spin coating using oligoEDOT-PCL polymer solutions. Namely, diEDOT-PCL, tetraEDOT-PCL, pentaEDOT-PCL and control PCL polymer solutions, including high *M*_w_ PCL (70–90 kDa) and low *M*_w_ PCL (24 kDa) were spin coated at 6 w/v %. Polymer solutions were prepared in CHCl_3_, sonicated for 15 min and left overnight to aid polymer dissolution. For UV–vis, microscope glass slides (10 mm × 10 mm) were cut with a diamond tip. The glass slides were cleaned by sonicating for 15 min each in acetone and isopropanol, dried under a nitrogen stream and treated with oxygen plasma before spin coating. 100 μL of polymer solution was spin coated on glass substrates for 20 s at 1500 rpm and placed in a fume hood overnight.

*UV–Vis Spectroscopy*: Absorbance spectra were recorded with a Shimadzu UV-1601 UV/vis spectrophotometer, in the range between 300 and 900 nm. The spectra diEDOT-PCL, tetraEDOT-PCL and pentaEDOT-PCL films were measured, and film samples were attached with blue tac to the outside of the cuvette holder. In order to calculate the optical band gap energy, the longest absorption wavelength *λ*_onset_ was used, according to the following equation.^[80]^

(1) $$E_{g}=1242/\lambda_{\text{onset}}$$

*Cyclic Voltammetry*: Cyclic voltammograms were conducted using an Autolab PGSTAT101 potentiostat. Recordings were carried out using a conventional three-electrode setup, using an isolated Ag/AgCl reference, platinum counter electrode, and a glassy carbon working electrode. Films were deposited on the glassy carbon electrode via drop casting. The measurements were carried out in 0.5 M tetrabutylammonium hexafluorophosphate dissolved in propylene carbonate as the supporting electrolyte. Samples were measured over the potential range of −0.45 to 1.20 V at a scan rate of 100 mV s^−1^.

*SEM*: OligoEDOT-PCL solution electrospun and MEW scaffolds were mounted on a SEM pin mount, and sputter coated with a thin gold layer. Images were obtained using a JSM 6010LA SEM (JEOL). NSCs on oligoEDOT-PCL scaffolds were pre-fixed with 3.7% (w/v) paraformaldehyde and washed in 0.1 M sodium cacodylate buffer for 5 min. NSCs were then fixed in 2.5% (w/v) glutaraldehyde in 0.1 M cacodylate buffer for 1 h at room temperature, and washed 2 } 5 min in 0.1 M sodium cacodylate buffer. For osmium tetroxide staining, a solution of 1% (v/v) OsO_4_ was prepared in 0.1 M sodium cacodylate buffer, and samples were incubated for 1 h. Samples were rinsed with milli-Q water twice for 5 min. Samples were then serially dehydrated in graded series of ethanol, as described, and treated with hexamethyldizilazane for 5 min. Samples were then attached to SEM pin mount and coated with 24 nm chromium in a sputter coater (Q150T S Quorum). Images were obtained using a Zeiss Sigma 300.

*AFM*: AFM measurements were performed on an Agilent 5500 AFM. Topographical images were obtained in tapping mode using Micromasch HQ-NSC cantilevers (nominal spring constant of 40 N m^−1^) at a resonant frequency of 260 kHz. Images were taken at 40, 10, and 2 μm scale, and were processed using Gwyddion software.

*KPFM*: KPFM was used to obtain information about the surface potential of the oligoEDOT-PCL films, and was performed using an Asylum MFP-3d microscope. Images were obtained using Nanosensors PPP-EFM cantilevers (nominal spring constant of 2.8 N m^−1^), coated in Pt/Ir with an applied DC bias of 0.2 V. CPD on the sample was calculated using the following equation

(2) $$\text{CPD}=\frac{\phi_{\text{tip}}-\phi_{\text{sample}}}{e}$$

where *e* is the electron charge. OligoEDOT-PCL samples were spincoated on ITO glass.

*Cell Culture*: Human episomal iPSC line (Epi-hiPSC) (Thermo Fisher Scientific, U.K.), were maintained in feeder-free conditions on Matrigel substrate with Essential 8 media (Thermo Fisher Scientific) and passaged when they reached 80–90% confluence with EDTA solution (0.5 mM EDTA/PBS). Neural induction was based on a previously published protocol.^[62]^ iPSCs were differentiated into neuroectoderm when they reached 80–90% confluence by dual SMAD signaling inhibition using neural induction medium [(Advanced DMEM/F-12 medium (Thermo Fisher Scientific), 0.2% (v/v) B27 Supplement (Invitrogen), 1% (v/v) N2 supplement (Invitrogen, U.K.), 1% (v/v) penicillin/streptomycin (Invitrogen), dorsomorphin (2 μM; Calbiochem, U.K.), 1% (v/v) GlutaMAX (Invitrogen) supplemented with SB431542 (10 μM; Tocris, U.K.), and N-acetylcysteine (1 mM; Sigma-Aldrich)] for 7 d, as previously described.^[62]^ NSCs were then passaged and plated on laminin-coated plates NSCR base medium^[62]^ (DMEM/F-12 medium (Thermo Fisher Scientific), 1% (v/v) N2 supplement, 0.2% B27 supplement, 1% (v/v) nonessential amino acids, 1% (v/v) penicillin/streptomycin and 1% (v/v) GlutaMax solution (all from Invitrogen), and B-27 medium (Neurobasal medium (Thermo fisher scientific), 2% (v/v) B27 supplement, 1% (v/v) nonessential amino acids, 1% (v/v) penicillin/streptomycin and 1% (v/v) GlutaMAX solution). After 3–5 days, iPSC derived NSCs formed neural rosette structures, and NSCs were then maintained in F20 medium (NSCR neural maintenance base medium supplemented with 20 ng mL^−1^ of FGF2 [PeproTech]) to promote expansion of NSCs. NSCs were typically passaged every 5 days on matrigel coated plates, and the medium was changed every 48 h, until cells reached 80–90% confluence.

*FILM Preparation for Cell Culture and Cell Culture Device Setup*: TetraEDOT-PCL was blended with high molecular weight PCL (*M*_w_ ≈ 75 000) at a 50:50 ratio and dissolved at 6% (w/v) in chloroform. Polymer films were prepared by spin coating on cover glass as described above and transferred into coverslip bottom 24 well plates (ibidi). Silicone O-rings (10.77 mm diameter) were autoclaved, placed over glass coverslips in the 24 well plates, followed by three washes in sterile PBS and sterilized by cell culture-grade UV-light irradiation for 30 min. 500 μL of matrigel (at a dilution of 1:120 in DMEM/F12 medium) was added to each well and incubated for 30 min at 37°C in a cell culture incubator. For electrical stimulation experiments, a custom-made cell culture device was designed using teflon microchambers, which were autoclaved prior to use. Polymer films were prepared by spin coating 6% (w/v) TetraEDOT-PCL on ITO glass electrodes, as described, and assembled in the microchambers in a cell culture hood. The wells were washed three times in sterile PBS and sterilized by cell culture-grade UV light irradiation for 30 min followed by 500 μL of matrigel (at a dilution of 1:120 in DMEM/F12 medium). NSCs were seeded on the polymer film samples at a density of 10 × 10^4^ per well using a dry plating method and left to incubate at 37°C for 20 min. For NSC proliferation and differentiation experiments, F20 media and 50:50 media was added, respectively, and medium was changed every 48 h. For electrical stimulation experiments, 50:50 media was added to promote neuronal differentiation.

*Electrical Stimulation of NSCs on OligoEDOT-PCL Films*: After 24 h of NSC seeding for cell attachment, a counter electrode platinum wire was suspended into the microchamber well, parallel to the oligoEDOT-PCL film seeded with NSCs. The platinum wire and ITO were connected to an eDaq potentiostat (EA163) and trains of 1 ms electrical pulses of 600 mV at 1 Hz were applied for a period of 24 h. After stimulation, NSCs were fixed with 3.7% (v/v) paraformaldehyde for 15 min followed by immunostaining.

*Immunostaining*: For NSC proliferation and differentiation experiments on oligoEDOT-PCL films, cells were washed with PBS and fixed with 3.7% (v/v) paraformaldehyde (Sigma-Aldrich) for 15 min at room temperature, and then washed three times with sterile PBS. OligoEDOT-PCL films were permeabilized with 0.2% (v/v) Triton X-100 (Sigma-Aldrich) in PBS for 10 min, and then blocked with 3% (v/v) goat serum (Sigma-Aldrich) for 30 min, followed with primary antibodies, nestin (1:500; Millipore, U.K.), *β*III-tubulin (1:1000; Sigma-Aldrich), and Ki67 (1:1000; Abcam), followed with DAPI (Sigma-Aldrich) and secondary antibodies (Alexa Fluor dyes; Thermo Fisher Scientific) for 1 h. For electrical stimulation experiments, cells were fixed, permeabilized and blocked as described, followed with primary antibodies, nestin (1:500; Millipore, U.K.), *β*III-tubulin (1:1000; Sigma-Aldrich), followed with DAPI (Sigma-Aldrich) and secondary antibodies (Alexa Fluor dyes; Thermo Fisher Scientific) for 1 h. For electrical stimulation experiments, stained samples on ITO glass were mounted with coverslip slides with FluorSave Reagent (Millipore) and stored at 4°C. Images of NSCs on polymer films were obtained with Inverted Widefield Microscope (Zeiss Axio Observer), and *Z* stacks of ≈30 slices were obtained. Images on electrospun fibrous mats were acquired with a Zeiss LSM 510 inverted confocal microscope.

*Imaging Analysis and Statistical Analysis*: Images analysis was performed with ImageJ software. *Z* stack images were deconvolved using Huygens software. Neural stem cell (NSC) proliferation and differentiation was analyzed by counting the percentage of *β*III-tubulin^+^ cells over the total number of cells, using the cell counter plugin. Neurite outgrowth was analyzed using the Neurite tracings plugin. Neurite outgrowth was analyzed by measuring individual neurites for each soma, using the Neurite tracings plugin in Fiji. Neurite branching was quantified using the cell counter plugin in Fiji. 3 or 4 random images were taken for each sample. For statistical analysis all experiments were conducted at least four times. Biocompatibility experiments were conducted with a minimum of 2 biological replicates and 3 technical replicates, and a minimum of 15 images were analyzed for each condition. Neurite length and branching experiments were conducted with a minimum of 3 biological replicates and 2 technical replicates, except where replicates without quantifiable cells were excluded from the analysis. A minimum of 12 images were analyzed for each condition. One-way ANOVA with post hoc Tukey’s test was used, and a *p*-value of <0.05 was considered statistically significant.

*Solution Electrospinning*: TetraEDOT-PCL was blended with high molecular weight PCL (*M*_w_ ≈ 75 000 Da) at a 50:50 ratio and dissolved at 20% (w/v) in a 9:1 mixture of chloroform and methanol. OligoEDOT-PCL polymer solutions were processed into fibrous scaffolds using a custom build electrospinning device, as previous described.^[57]^ Scaffolds were spun at 12 kV, 20 μL min^−1^ flow rate, with a distance of 15 cm between the needle and collector plate. Fiber diameters were measured using ImageJ, based on SEM.

*MEW*: TetraEDOT-PCL was blended with PCL (*M*_w_ ≈ 75 000 Da) at a 50:50 ratio, by dissolving in CHCl_3_, and precipitating in diethyl ether. The blended polymer was loaded into a 3 cc polypropylene syringe (Nordson #7 012 074) and heated overnight at 75°C in a vacuum oven to remove bubbles. A 23G needle (Nordson #7 018 302) was installed, and the syringe was inserted into the spinneret. After equilibrating for 30 min at 70°C, scaffolds were printed using a 10 kV accelerating voltage, 10 mm collector distance, axis velocity of 1500 mm min^−1^, feeding air pressure of 1.0 Bar, and heating temperatures between 60 and 80°C. Printing was controlled by MACH 3 CNC software (ARTSOFT, Livermore Falls, USA), and dimensions were specified by G-code. Each scaffold consisted of 10 stacked layers.

Scaffolds were detached from the collector plate using a drop of ethanol and moved to a petri-dish. For cell culture experiments, scaffolds were sterilized by cell culture-grade UV light irradiation for 30 min.

## Supplementary Material

Supporting Information is available from the Wiley Online Library or from the author.

Supplementary Information

## References

[1] Hopkins AM, DeSimone E, Chwalek K, Kaplan DL (2015). Prog Neurobiol.

[2] Boni R, Ali A, Shavandi A, Clarkson AN (2018). J Biomed Sci.

[3] Bosi S, Rauti R, Laishram J, Turco A, Lonardoni D, Nieus T, Prato M, Scaini D, Ballerini L (2015). Sci Rep.

[4] Baklaushev VP, Bogush VG, Kalsin VA, Sovetnikov NN, Samoilova EM, Revkova VA, Sidoruk KV, Konoplyannikov MA, Timashev PS, Kotova SL, Yushkov KB (2019). Sci Rep.

[5] Wang Z-H, Chang Y-Y, Wu J-G, Lin C-Y, An H-L, Luo S-C, Tang TK, Su W-F (2018). Macromol Biosci.

[6] Ghasemi-Mobarakeh L, Prabhakaran MP, Morshed M, Nasr-Esfahani MH, Baharvand H, Kiani S, Al-Deyab SS, Ramakrishna S (2011). J Tissue Eng Regener Med.

[7] Meco E, Lampe KJ (2018). Front Mater.

[8] Ghasemi-Mobarakeh L, Prabhakaran MP, Morshed M, Nasr-Esfahani MH, Ramakrishna S (2010). Mater Sci Eng, C.

[9] Yang F, Murugan R, Wang S, Ramakrishna S (2005). Biomaterials.

[10] Seidlits SK, Lee JY, Schmidt CE (2008). Nanomedicine.

[11] D’Amato AR, Puhl DL, Ziemba AM, Johnson CDL, Doedee J, Bao J, Gilbert RJ (2019). PLoS One.

[12] Prabhakaran MP, Venugopal JR, Ter Chyan T, Hai LB, Chan CK, Lim AY, Ramakrishna S (2008). Tissue Eng, Part A.

[13] Corey JM, Lin DY, Mycek KB, Chen Q, Samuel S, Feldman EL, Martin DC (2007). J Biomed Mater Res, Part A.

[14] Lee JY, Bashur CA, Gomez N, Goldstein AS, Schmidt CE (2010). J Biomed Mater Res, Part A.

[15] Jakobsson A, Ottosson M, Zalis MC, O’Carroll D, Johansson UE, Johansson F (2017). Nanomedicine Nanotechnology, Biol Med.

[16] Kim D, Kim S-M, Lee S, Yoon M-H (2017). Sci Rep.

[17] Soliman E, Bianchi F, Sleigh JN, George JH, Cader MZ, Cui Z, Ye H (2018). Biotechnol Lett.

[18] Brown TD, Dalton PD, Hutmacher DW (2011). Adv Mater.

[19] Muerza-Cascante ML, Haylock D, Hutmacher DW, Dalton PD (2014). Tissue Eng, Part B Rev.

[20] Schmidt CE, Shastri VR, Vacanti JP, Langer R (1997). Proc Natl Acad Sci USA.

[21] Patel N, Poo M-M (1982). J Neurosci.

[22] McCaig CD, Song B, Rajnicek AM (2009). J Cell Sci.

[23] Levin M, Pezzulo G, Finkelstein JM (2017). Annu Rev Biomed Eng.

[24] Green R, Abidian MR (2015). Adv Mater.

[25] Guimard NK, Gomez N, Schmidt CE (2007). Prog Polym Sci.

[26] Deslouis C, El Moustafid T, Musiani MM, Tribollet B (1996). Electro-chim Acta.

[27] Alegret N, Dominguez-Alfaro A, Mecerreyes D (2019). Biomacromolecules.

[28] Zhang Q, Beirne S, Shu K, Esrafilzadeh D, Huang X-F, Wallace GG (2018). Sci Rep.

[29] Vijayavenkataraman S, Kannan S, Cao T, Fuh JYH, Sriram G, Lu WF (2019). Front Bioeng Biotechnol.

[30] Wang Z, Wang Z, Lu WW, Zhen W, Yang D, Peng S (2017). NPG Asia Mater.

[31] Tran H, Feig VR, Liu K, Wu H-C, Chen R, Xu J, Deisseroth K, Bao Z (2019). ACS Cent Sci.

[32] Green RA, Hassarati RT, Goding JA, Baek S, Lovell NH, Martens PJ, Poole-Warren LA (2012). Macromol Biosci.

[33] Baek S, Green RA, Poole-Warren LA (2014). J Biomed Mater Res, Part A.

[34] Caliari SR, Burdick JA (2016). Nat Methods.

[35] El-Sherbiny IM, Yacoub MH (2013). Glob Cardiol Sci Pract.

[36] Spicer CD (2020). Polym Chem.

[37] Goding J, Gilmour A, Martens P, Poole-Warren L, Green R (2017). Adv Healthc Mater.

[38] Mario Cheong GL, Lim KS, Jakubowicz A, Martens PJ, Poole-Warren LA, Green RA (2014). Acta Biomater.

[39] Zhou Y, Wan C, Yang Y, Yang H, Wang S, Dai Z, Ji K, Jiang H, Chen X, Long Y (2019). Adv Funct Mater.

[40] Goding J, Green R, Martens P, Poole-Warren L (2015). RSC Smart Mater.

[41] Aregueta-Robles UA, Woolley AJ, Poole-Warren LA, Lovell NH, Green RA (2014). Front Neuroeng.

[42] Guo B, Glavas L, Albertsson A-C (2013). Prog Polym Sci.

[43] Lin Y, Zhan X (2016). Acc Chem Res.

[44] Wang Y, Tran HD, Kaner RB (2011). Macromol Rapid Commun.

[45] Liu K, Liu B (2018). Biomacromolecules.

[46] Heinrich MA, Liu W, Jimenez A, Yang J, Akpek A, Liu X, Pi Q, Mu X, Hu N, Schiffelers RM, Prakash J (2019). Small.

[47] Rivers TJ, Hudson TW, Schmidt CE (2002). Adv Funct Mater.

[48] Lee H, Gu L, Mooney DJ, Levenston ME, Chaudhuri O (2017). Nat Mater.

[49] Madl CM, Lesavage BL, Dewi RE, Dinh CB, Stowers RS, Khariton M, Lampe KJ, Nguyen D, Chaudhuri O, Enejder A, Heilshorn SC (2017). Nat Mater.

[50] Hardy JG, Mouser DJ, Arroyo-Currás N, Geissler S, Chow JK, Nguy L, Kim JM, Schmidt CE (2014). J Mater Chem B.

[51] Guimard NKE, Sessler JL, Schmidt CE (2009). Macromolecules.

[52] Xu C, Huang Y, Yepez G, Wei Z, Liu F, Bugarin A, Tang L, Hong Y (2016). Sci Rep.

[53] Woodard LN, Grunlan MA (2018). ACS Macro Lett.

[54] Spicer CD, Booth MA, Mawad D, Armgarth A, Nielsen CB, Stevens MM (2017). Chem.

[55] Carsten B, He F, Son HJ, Xu T, Yu L (2011). Chem Rev.

[56] Park B, Yang L, Johansson EMJ, Vlachopoulos N, Chams A, Perruchot C, Jouini M, Boschloo G, Hagfeldt A (2013). J Phys Chem C.

[57] Chen Y, Gan T, Ma C, Wang L, Zhang G (2016). J Phys Chem B.

[58] Guex AG, Spicer CD, Armgarth A, Gelmi A, Humphrey EJ, Terracciano CM, Harding SE, Stevens MM (2017). MRS Commun.

[59] Spicer CD, Pashuck ET, Stevens MM (2018). Chem Rev.

[60] Lutolf MP, Tirelli N, Cerritelli S, Cavalli L, Hubbell JA (2001). Bioconjug Chem.

[61] Darling NJ, Hung YS, Sharma S, Segura T (2016). Biomaterials.

[62] Jansen LE, Negrón-Piñeiro LJ, Galarza S, Peyton SR (2018). Acta Biomater.

[63] Bhagwat N, Murray RE, Shah SI, Kiick KL, Martin DC (2016). Acta Biomater.

[64] Mawad D, Artzy-Schnirman A, Tonkin J, Ramos J, Inal S, Mahat MM, Darwish N, Zwi-Dantsis L, Malliaras GG, Gooding JJ, Lauto A (2016). Chem Mater.

[65] Yano H, Kudo K, Marumo K, Okuzaki H (2019). Sci Adv.

[66] Chambers SM, Fasano CA, Papapetrou EP, Tomishima M, Sadelain M, Studer L (2009). Nat Biotechnol.

[67] Hsu C-C, Serio A, Amdursky N, Besnard C, Stevens MM (2018). ACS Appl Mater Interfaces.

[68] Lannutti J, Reneker D, Ma T, Tomasko D, Farson D (2007). Mater Sci Eng, C.

[69] Pedersen JA, Swartz MA (2005). Ann Biomed Eng.

[70] Soman P, Tobe BTD, Lee JW, Winquist AM, Singec I, Vecchio KS, Snyder EY, Chen S (2012). Biomed Microdevices.

[71] Wong JY, Langer R, Ingber DE (1994). Proc Natl Acad Sci USA.

[72] Jager EWH, Bolin MH, Svennersten K, Wang X, Richter-Dahlfors A, Berggren M (2009).

[73] Tanamoto R, Shindo Y, Miki N, Matsumoto Y, Hotta K, Oka K (2015). J Neurosci Methods.

[74] Stewart E, Kobayashi NR, Higgins MJ, Quigley AF, Jamali S, Moulton SE, Kapsa RM, Wallace GG, Crook JM (2015). Tissue Eng, Part C.

[75] Pires F, Ferreira Q, Rodrigues CAV, Morgado J, Ferreira FC (2015). Biochim Biophys Acta, Gen Subj.

[76] Zhu B, Luo S-C, Zhao H, Lin H-A, Sekine J, Nakao A, Chen C, Yamashita Y, Yu H-H (2014). Nat Commun.

[77] Abidian MR, Corey JM, Kipke DR, Martin DC (2010). Small.

[78] Seegers J, Engelbrecht C, va Papendorp DH (2001). Med Hypotheses.

[79] Kotwal A, Schmidt CE (2001). Biomaterials.

[80] Leonat L, Sbârcea G, Brânzoi IV (2013). UPB Sci Bull, Ser B.

