# Supplementary material for: An Electroactive Oligo-EDOT Platform for Neural Tissue Engineering
Source: Adv Funct Mater. Author manuscript; Available in PMC 2021 May 24. (PMC7610826; doi:10.1002/adfm.202003710)
Supplement: Supplementary Information [file EMS103714-supplement-Supplementary_Information.pdf]

© 2020 Wiley-VCH GmbH

ADVANCED FUNCTIONAL MATERIALS

Supporting Information

for *Adv. Funct. Mater.,* DOI: 10.1002/adfm.202003710

An Electroactive Oligo-EDOT Platform for Neural Tissue Engineering

*Kaja I. Ritzau-Reid, Christopher D. Spicer, Amy Gelmi, Christopher L. Grigsby, James F. PonderJr., Victoria Bemmer, Adam Creamer, Ramon Vilar, Andrea Serio, and Molly M. Stevens**

Copyright WILEY-VCH Verlag GmbH & Co. KGaA, 69469 Weinheim, Germany, 2020.

Supporting Information

Title: An electroactive oligo-EDOT platform for neural tissue engineering

*Kaja I. Ritzau-Reid, Christopher D. Spicer, Amy Gelmi, Christopher L. Grigsby, James F. Ponder Jr., Victoria Bemmer, Adam Creamer, Ramon Vilar, Andrea Serio, Molly M. Stevens**

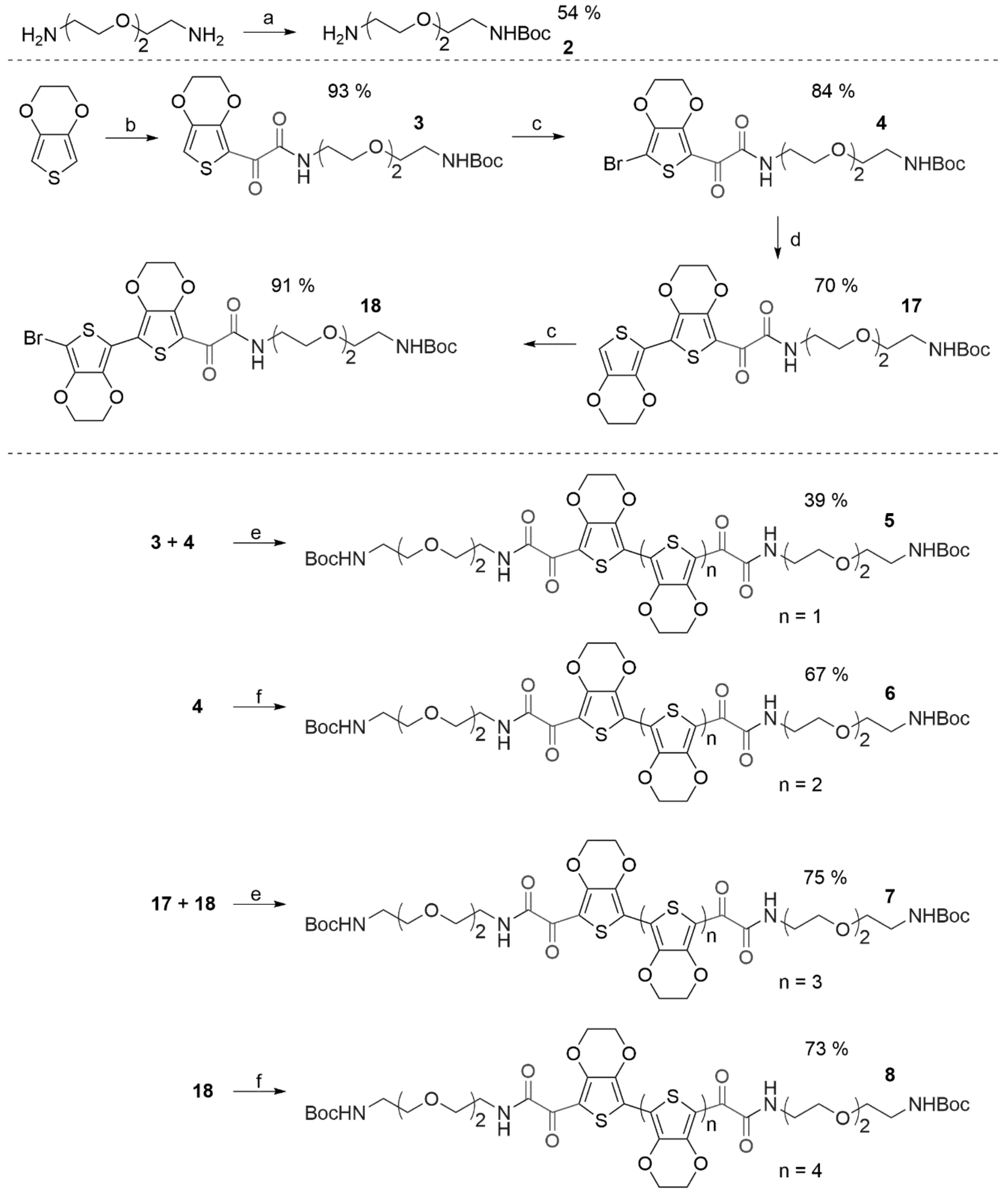

a) Boc_2O, DCM; b) i) $(COCl)_2$, Dioxane, ii) **2**; c) NBS, DMF; d) EDOT (4 equiv.), PivOH, $Pd(OAc)_2$, K_2CO_3, DMF, 90 °C; e) PivOH, $Pd(OAc)_2$, K_2CO_3, DMF, 90 °C; d) EDOT (0.55 equiv.), PivOH, $Pd(OAc)_2$, K_2CO_3, DMF, 90 °C;

Figure S1. Core oligoEDOT synthesis scheme.

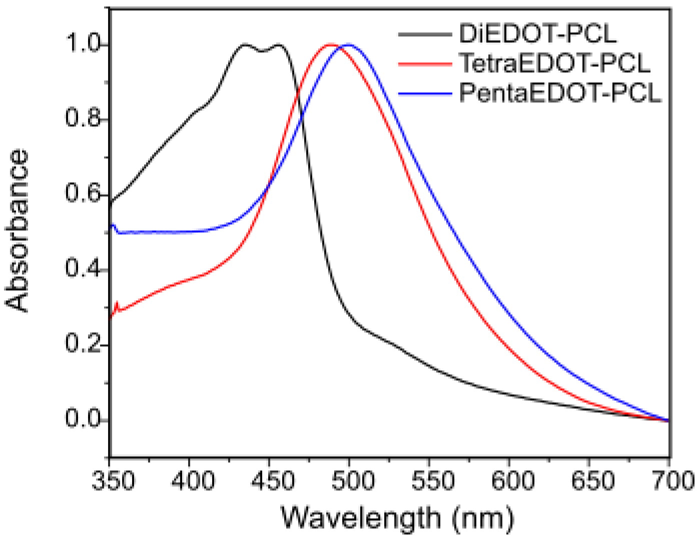

Figure S2. Normalized UV-Vis spectra of oligoEDOT-PCL polymers.

Table S1. Optical properties of oligoEDOT-PCL.

Polymer	λmax (nm)	Eg (eV)
DiEDOT-PCL	435, 456	2.48
TetraEDOT-PCL	490	2.09
PentaEDOT-PCL	500	2.03

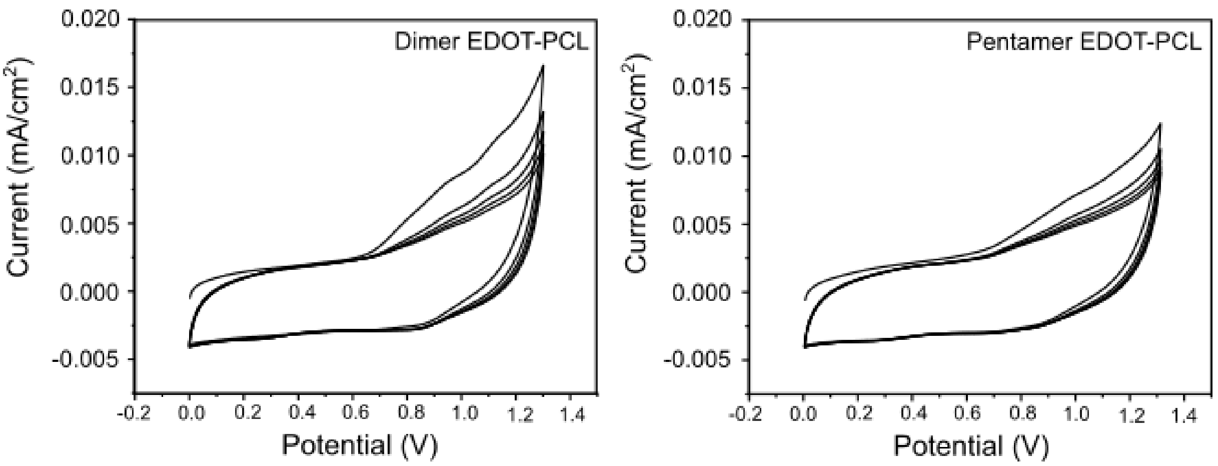

Figure S3. Cyclic voltammograms (100 mV/s) of dimer and pentamer oligoEDOT-PCL films in propylene carbonate with 0.5 M tetrabutylammonium hexafluorophosphate as the supporting electrolyte.

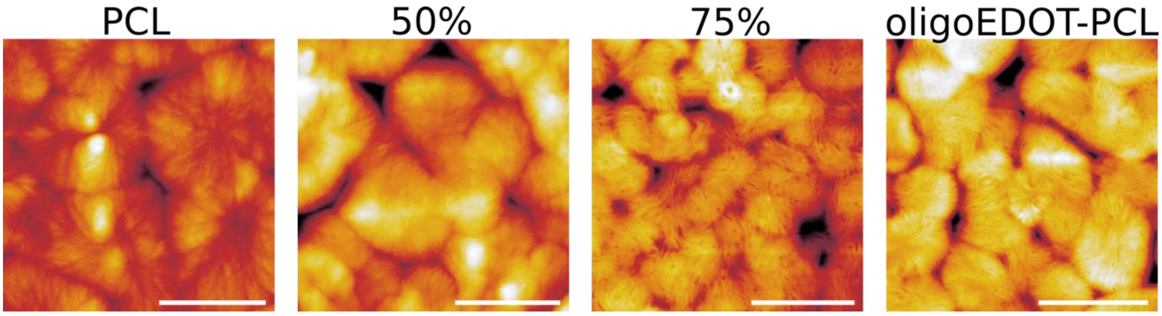

Figure S4. AFM topography images of pure PCL (~75 kDa) and PCL blended with indicated percentages of tetramer oligoEDOT-PCL and unblended tetramer oligoEDOT-PCL. Scale bar: 4 µm.

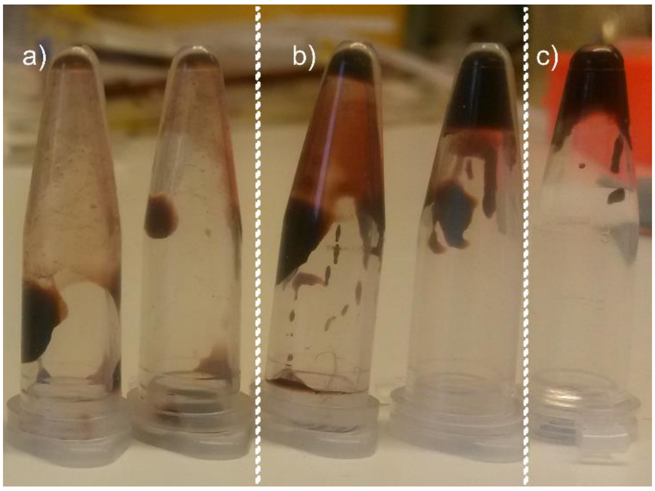

Figure S5. Representative images of attempted hydrogelation. Gels were formed in Eppendorf tubes, left for 15 minutes, inverted, and agitated. a) Amino-trimer **10** and NHS ester-capped PEG. Oligomer precipitation rapidly occurred and no gel was formed; b) Cys-capped trimer **26** and maleimide-capped PEG. Partial gelation occurred, but low water solubility led to mechanically weak gels; c) Cys-Glu-Glu-capped trimer **15** and maleimide-capped PEG. Gels were mechanically strong.

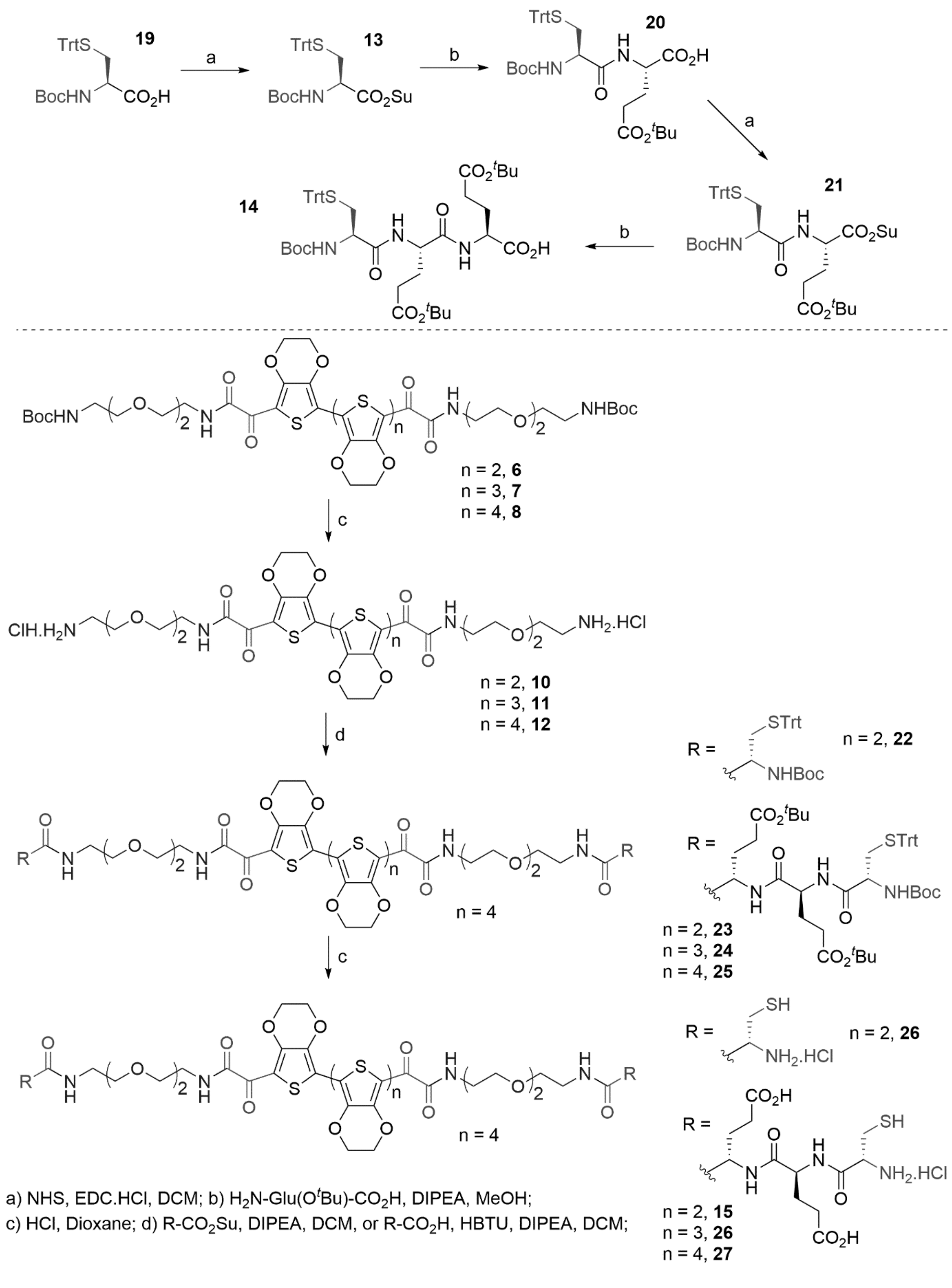

Figure S6. Synthesis of oligoEDOT hydrogelators.

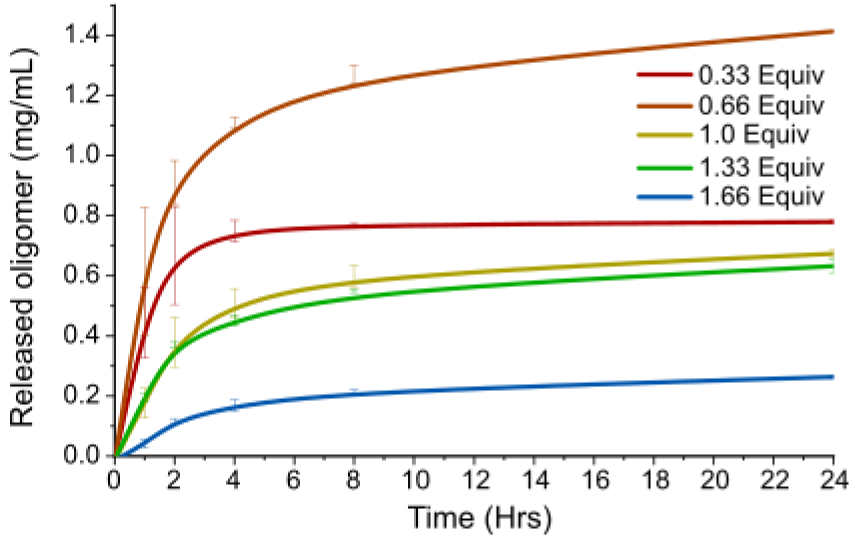

Figure S7. Cumulative release profile of oligomer **15** from hydrogels formed with different thiol:maleimide ratios.

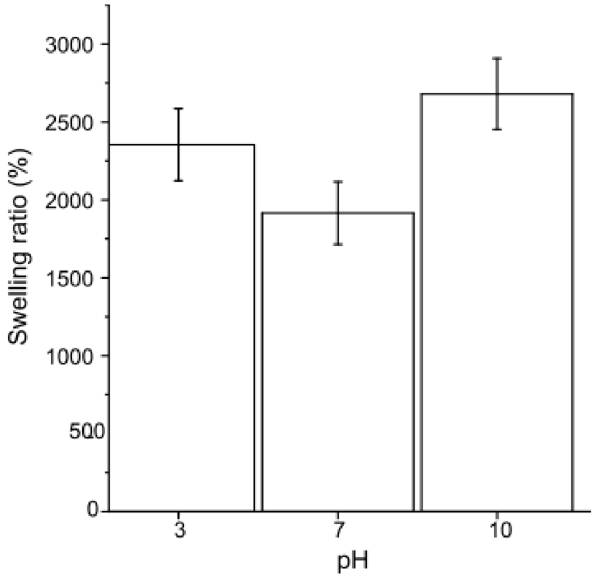

Figure S8. Swelling ratios of trimer **15** crosslinked gels at different pHs. N = 3.

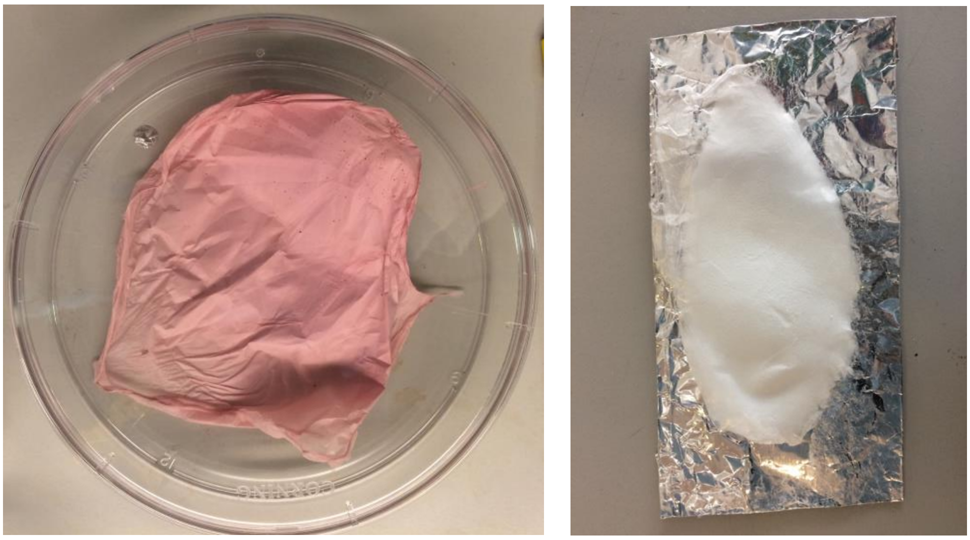

Figure S9. Solution electrospun tetraEDOT-PCL scaffold (left) and PCL scaffold (right) (petri dish dimeter 10 cm for comparison).

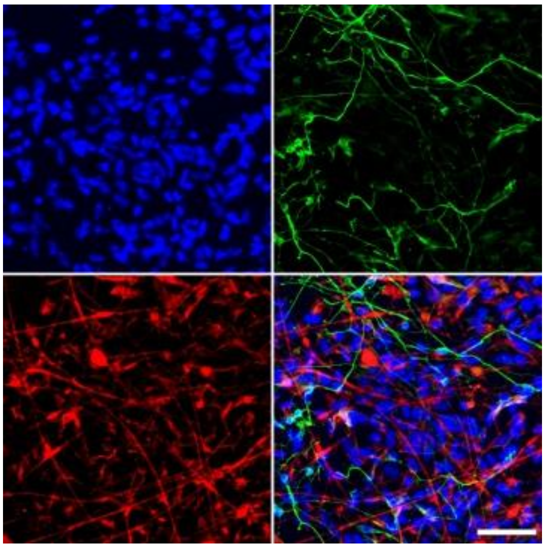

Figure S10. Biocompatibility of solution electrospun oligoEDOT-PCL scaffolds for neuronal cell culture. NSCs stained with βIII-tubulin (green), a neuronal marker, and nestin (red), a neural stem cell marker after 24 hours of neuronal differentiation. Scale bar: 50 µm.

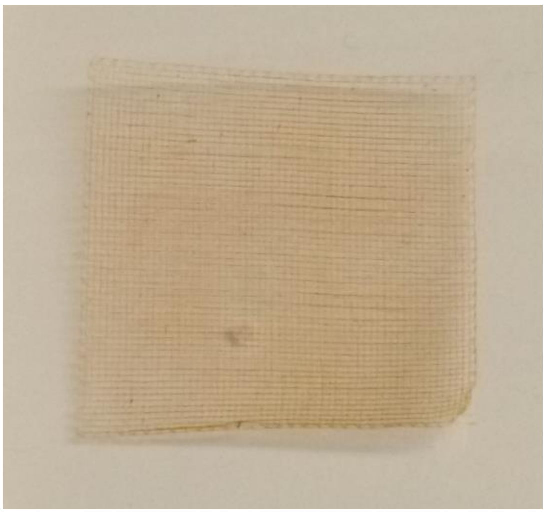

Figure S11. Melt electrospun writing (MEW) tetraEDOT-PCL scaffold (10 x 10 cm).

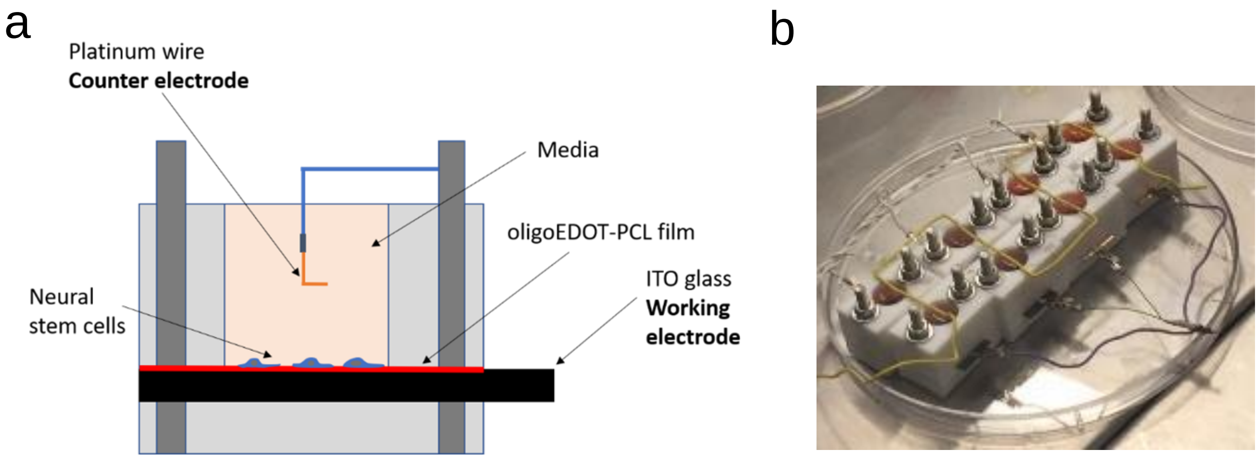

Figure S12. Electrical stimulation (ES) setup. (a) Schematic of cell culture device for ES. (b) Photograph of custom-made Teflon constructs setup with platinum wire counter electrode for ES.

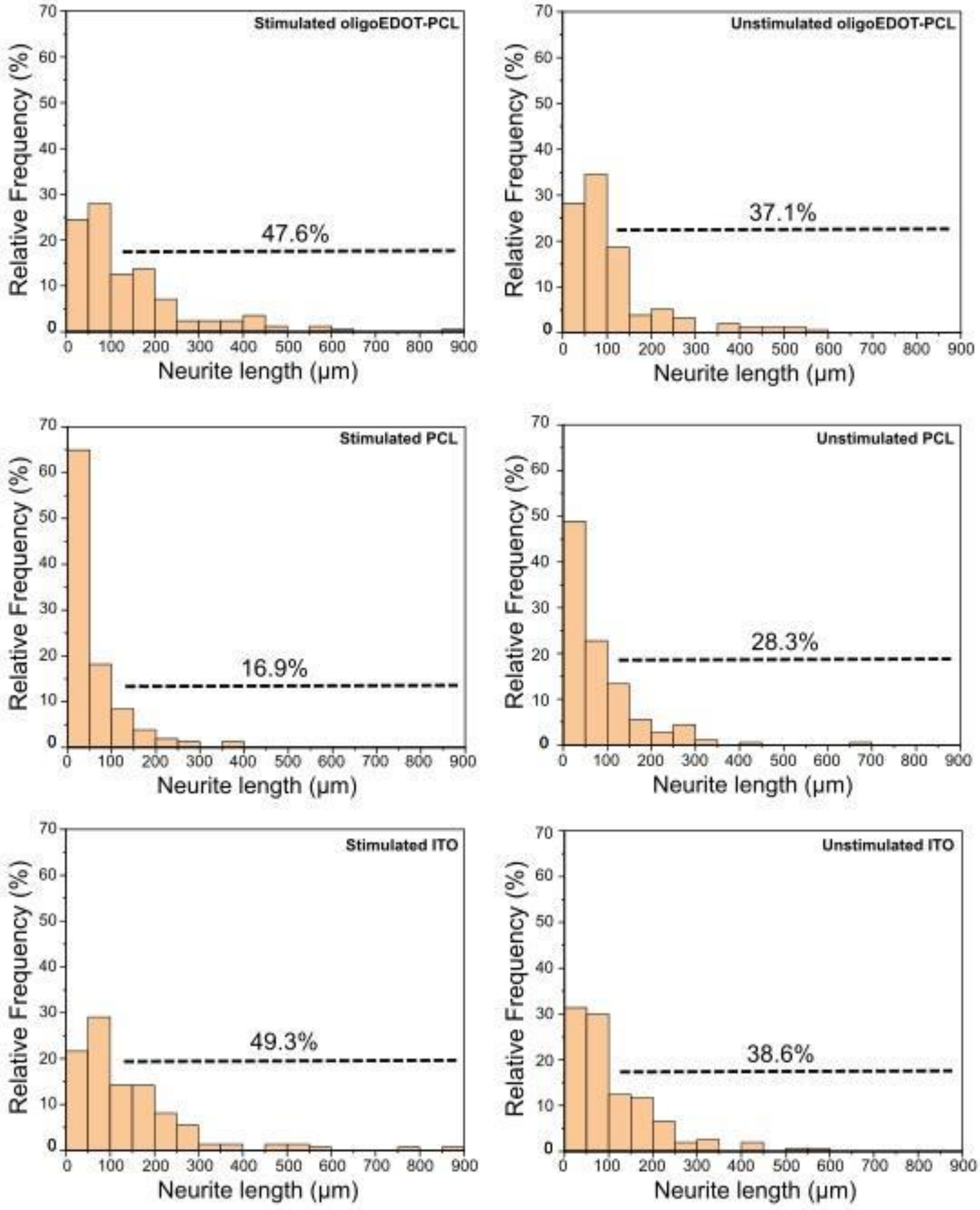

Figure S13. Frequency distribution histograms of neurite length on stimulated and unstimulated substrates ITO glass, tetraEDOT-PCL and PCL. Percentage of neurites that are over 100 µm as indicated by dashed line after 24 hours stimulation.

Core EDOT-oligomer synthesis

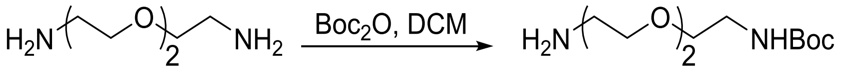

A solution of di-*tert*-butyl decarbonate (2.3 g, 10.5 mmol) in DCM (200 mL) was added dropwise over ~ 2 hrs to a solution of 2,2′-(ethylenedioxy)bis(ethylamine) (7.6 mL, 52.7 mmol) in DCM (50 mL). After stirring for 18 hrs, the mixture was washed with water (4 x 150 mL) and brine (100 mL), dried with MgSO4, filtered, and concentrated in vacuo to give the DP as a colourless oil. A yield of 1.4 g, 5.6 mmol (54 %) was obtained. Spectroscopic data were consistent with those previously reported.[1] ^{1}H NMR (400 MHz, $CDCl_3$): δ = 5.21 (1H, br s, -N<u>H</u>Boc), 3.53-3.64 (4H, m, -C<u>H</u>$_2$-), 3.45-3.54 (4H, m, -C<u>H</u>$_2$-), 3.28 (2H, dt, $J_1 = J_2 = 5.3$ Hz, -C<u>H</u>$_2$NHBoc), 2.84 (2H, t, $J = 5.2$ Hz, -C<u>H</u>$_2$NH$_2$), 1.73 (2H, br s, -N<u>H</u>$_2$), 1.40 (9H, s, <u>Boc</u>) ppm;

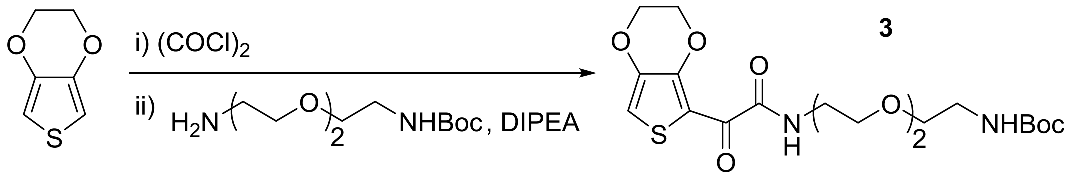

Oxalyl chloride (1.2 mL, 14 mmol) was added drop-wise to a solution of EDOT (1.5 mL, 14 mmol) in dioxane (30 mL). The mixture was heated to 100 ºC for 1 h then allowed to cool to room temperature. The resultant yellow solution was added drop-wise over ~ 5 min to a solution of amine **2** (4.96 g, 20 mmol) and DIPEA (9.7 mL, 56 mmol) in DCM (100 mL). The mixture was stirred for 4 hrs then diluted with DCM (150 mL), washed with water (100 mL), and the organics dried with $MgSO_4$, filtered and concentrated *in vacuo*. The residue was purified by flash column chromatography eluting with 80-100 % EtOAc:Hexane. Pure fractions were concentrated in vacuo to give the DP as a yellow oil. A yield of 5.2 g, 13 mmol (93 %) was obtained. ^{1}H NMR (400 MHz, $CDCl_3$): δ = 7.80 (1H, br s, -N<u>H</u>COCO-), 6.88 (1H, s, <u>H</u>5), 5.09 (1H, br s, -N<u>H</u>Boc), 4.41-4.47 (2H, m, ArC4-OC<u>H</u>$_2$-), 4.24-4.29 (2H, m, ArC3-OC<u>H</u>$_2$-), 3.60-3.67 (6H, m, -OC<u>H</u>$_2$-), 3.54-3.60 (4H, m, -OC<u>H</u>$_2$-), 3.34 (2H, dt, $J_1 = J_2 =$

4.8 Hz, -NHC<u>H</u>$_2$-), 1.45 (9H, s, <u>Boc</u>) ppm; ^{13}C NMR (100 MHz, CDCl$_3$): δ = 175.98 (-<u>C</u>OCONHR), 161.58 (-CO<u>C</u>ONHR), 156.00 (-<u>C</u>O$_2{}^t$Bu), 150.23 (Ar<u>C</u>4), 141.52 (Ar<u>C</u>3), 115.29 (Ar<u>C</u>5), 110.78 (Ar<u>C</u>2), 79.21 (-<u>C</u>Me$_3$), 70.37 (-O<u>C</u>H$_2$CH$_2$-), 70.22 (-O<u>C</u>H$_2$CH$_2$-), 69.37 (-O<u>C</u>H$_2$CH$_2$-), 65.56 (ArC4-O<u>C</u>H$_2$-), 63.89 (ArC3-O<u>C</u>H$_2$-), 40.37 (-NH<u>C</u>H$_2$CH$_2$-), 39.24 (-NH<u>C</u>H$_2$CH$_2$-), 20.41 (-C<u>Me</u>$_3$) ppm; IR (υ_{max}, film): 3353, 2975, 2932, 2871, 1685, 1638, 1523, 1473, 1449, 1424, 1363, 1270, 1249, 1173, 1101, 1071 cm^{-1}; HRMS m/z (ESI+): Found: 445.1635 (M+H), Calc.: 445.1645.

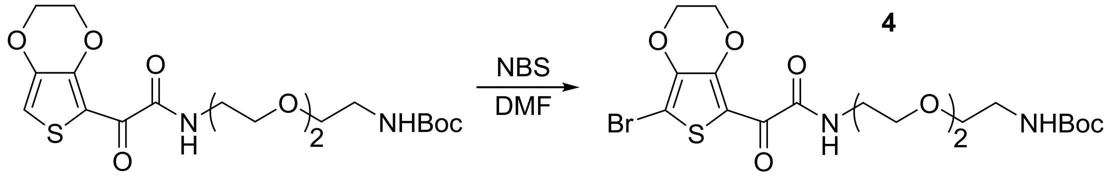

N-Bromosuccinimide (1.06 g, 6 mmol) was added to a solution of Boc-protected EDOT **3** (2.2 g, 5 mmol) in DMF (15 mL) in the dark. After stirring for 90 min, the mixture was diluted with EtOAc (150 mL). The organics were washed with 1 M $KHSO_4$ (100 mL) and brine (100 mL), dried with $MgSO_4$, filtered and concentrated *in vacuo*. The residue was purified by flash column chromatography eluting with 80-100 % EtOAc:Hexane. Pure fractions were concentrated *in vacuo* to give the DP as a yellow oil which solidified on standing. A yield of 2.19 g, 4.2 mmol (84 %) was obtained. ^{1}H NMR (400 MHz, CDCl$_3$): δ = 7.75 (1H, br s, -N<u>H</u>COCO-), 5.06 (1H, br s, -N<u>H</u>Boc), 4.42-4.46 (2H, m, ArC4-OC<u>H</u>$_2$-), 4.31-4.35 (2H, m, ArC3-OC<u>H</u>$_2$-), 3.58-3.65 (6H, m, -OC<u>H</u>$_2$-), 3.52-3.58 (4H, m, -OC<u>H</u>$_2$-), 3.33 (2H, dt, $J_1 = J_2$ = 5.1 Hz, -NHC<u>H</u>$_2$-), 1.43 (9H, s, <u>Boc</u>) ppm; ^{13}C NMR (100 MHz, CDCl$_3$): δ = 174.78 (-<u>C</u>OCONHR), 161.42 (-CO<u>C</u>ONHR), 155.97 (-<u>C</u>O$_2{}^t$Bu), 149.96 (Ar<u>C</u>4), 140.02 (Ar<u>C</u>3), 110.69 (Ar<u>C</u>2), 106.62 (Ar<u>C</u>5), 79.19 (-<u>C</u>Me$_3$), 70.35 (-O<u>C</u>H$_2$CH$_2$-), 70.20 (-O<u>C</u>H$_2$CH$_2$-), 69.27 (-O<u>C</u>H$_2$CH$_2$-), 65.54 (ArC4-O<u>C</u>H$_2$-), 64.35 (ArC3-O<u>C</u>H$_2$-), 40.35 (-NH<u>C</u>H$_2$CH$_2$-), 39.29 (-NH<u>C</u>H$_2$CH$_2$-), 28.41 (-C<u>Me</u>$_3$) ppm; IR (υ_{max}, solid): 3351, 2976, 2932, 2886, 1682, 1637, 1533, 1479, 1469, 1455, 1421, 1363, 1323, 1279, 1246, 1229, 1173, 1137, 1112, 1080 cm^{-1}; HRMS m/z (ESI+): Found: 523.0806/525.0785 (M+H), Calc.: 523.0793/525.0724.

General chain extension/oligomer coupling protocol

Brominated oligomer (1 mmol), hydrogen-capped oligomer (x equivalents), pivalic acid (0.5 mmol), palladium (II) acetate (0.05 mmol) and potassium carbonate (10 mmol) were charged under nitrogen. Dry DMF (2 mL) was added and the mixture heated to 90 ºC for 30 min. After cooling to rt the mixture was diluted with DCM (50 mL) and washed with water (2 x 50 mL) and brine (50 mL). The organics were dried with MgSO4, filtered and concentrated in vacuo. The residue was purified by flash column chromatography and pure fractions were concentrated *in vacuo*.

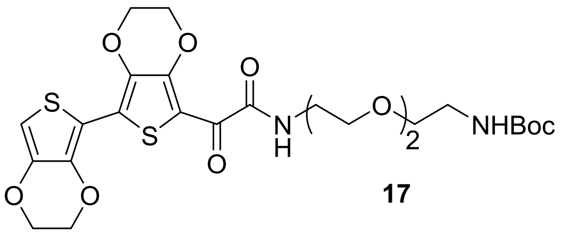

17

Run on 1.9 mmol scale with brominated monomer **4** and EDOT (4 equiv.). Column eluted with 70-100 % EtOAc:Hexane. Yield of 780 mg, 1.33 mmol (70 %) as an orange solid. ^{1}H NMR (400 MHz, $CDCl_3$): δ = 7.85 (1H, br s, -NHCOCO-), 6.50 (1H, s, H10), 5.10 (1H, br s, -NHBoc), 4.47-4.52 (2H, m, Ar-OCH_2-), 4.38-4.45 (4H, m, Ar-OCH_2-), 4.24-4.29 (2H, m, Ar-OCH_2-), 3.54-3.67 (10H, m, -OCH_2-), 3.37 (2H, dt, $J_1 = J_2$ = 4.9 Hz, -NHCH_2-), 1.45 (9H, s, Boc) ppm; ^{13}C NMR (100 MHz, $CDCl_3$): δ = 175.10 (-COCONH-), 162.19 (-COCONH-), 156.00 (-$CO_2{}^t$Bu), 149.99 (ArC_β), 141.41 (ArC_β), 140.73 (ArC_β), 136.26 (ArC_β), 126.26 (ArC_α), 109.35 (ArC_α), 108.15 (ArC_α), 102.29 (ArC10), 79.18 (-CMe_3), 70.41 (-OCH_2CH_2-), 70.37 (-OCH_2CH_2-), 70.23 (-OCH_2CH_2-), 69.47 (-OCH_2CH_2-), 65.56 (Ar-OCH_2-), 65.32 (Ar-OCH_2-), 64.52 (Ar-OCH_2-), 64.34 (Ar-OCH_2-), 40.38 (-NHCH_2CH_2-), 39.24 (-NHCH_2CH_2-), 28.42 (-CMe_3) ppm; IR (υ_{max}, solid): 3333, 2976, 2932, 2871, 1695, 1677, 1626, 1523, 1490, 1470, 1439, 1361, 1277, 1249, 1169, 1111, 1090 cm^{-1}; HRMS m/z (ESI+): Found: 585.1563 (M+H), Calc.: 585.1577.

200 mg, 0.4 mmol (20 %) of di-functional trimer **6** was also obtained as a red solid after eluting column with 10 % MeOH:EtOAc. Characterisation is provided below.

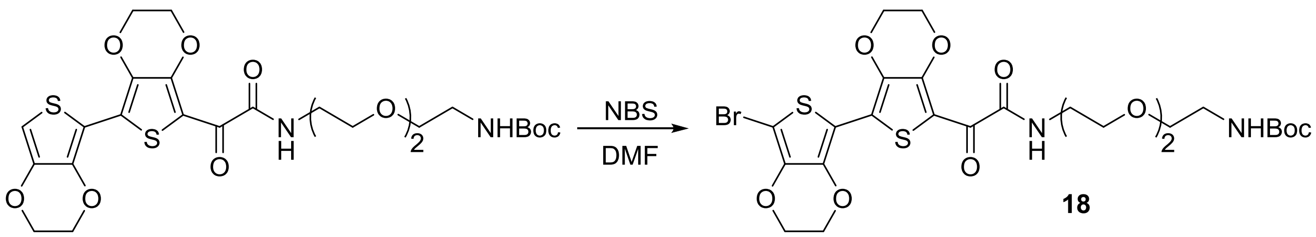

N-Bromosuccinimide (256 mg, 1.44 mmol) was added to a solution of Boc-protected EDOT **17** (700 mg, 1.2 mmol) in DMF (10 mL) in the dark. After stirring for 90 min, the mixture was precipitated in diethyl ether (150 mL). The precipitate was collected by filtration, washed with diethyl ether (50 mL), and dried *in vacuo* to give the DP as a red solid. A yield of 867 mg, 1.31 mmol (91 %) was obtained. ^{1}H NMR (400 MHz, $CDCl_3$): δ = 7.85 (1H, br s, -NHCOCO-), 5.01 (1H, br s, -NHBoc), 4.48-4.52 (2H, m, Ar-OCH_2-), 4.38-4.45 (4H, m, Ar-OCH_2-), 4.33-4.37 (2H, m, Ar-OCH_2-), 3.54-3.67 (10H, m, -OCH_2-), 3.34-3.39 (2H, m, -NHCH_2-), 1.46 (9H, s, Boc) ppm; ^{13}C NMR (100 MHz, $CDCl_3$): δ = 175.11 (-COCONH-), 162.11 (-COCONH-), 156.01 (-$CO_2{}^{t}$Bu), 149.85 (ArC_β), 139.89 (ArC_β), 139.67 (ArC_β), 136.33 (ArC_β), 125.11 (ArC_α), 109.24 (ArC_α), 108.28 (ArC_α), 91.24 (ArC10), 79.20 (-CMe_3), 70.41 (-OCH_2CH_2-), 70.38 (-OCH_2CH_2-), 70.23 (-OCH_2CH_2-), 69.44 (-OCH_2CH_2-), 65.57 (Ar-OCH_2-), 65.26 (Ar-OCH_2-), 64.98 (Ar-OCH_2-), 64.43 (Ar-OCH_2-), 40.39 (-NHCH_2CH_2-), 39.25 (-NHCH_2CH_2-), 28.43 (-CMe_3) ppm; IR (υ_{max}, solid): 3407, 3320, 2978, 2926, 2878, 1649, 1672, 1630, 1523, 1495, 1469, 1444, 1426, 1389, 1358, 1276, 1251, 1168, 1112, 1065 cm^{-1}; HRMS m/z (ESI+): Found: 663.0663/665.0594 (M+H), Calc.: 663.0682/665.0656.

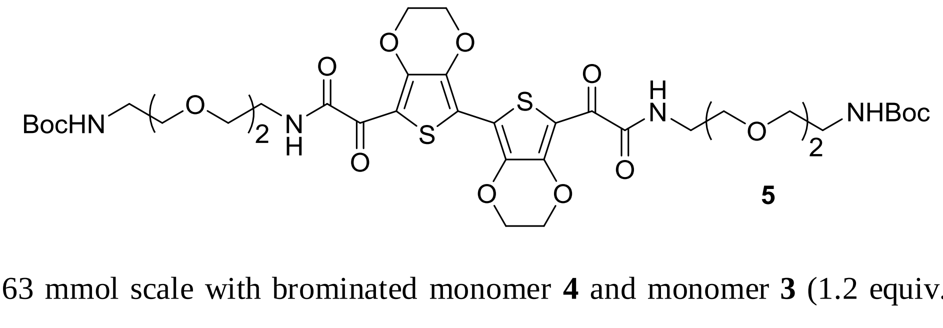

Run on 0.63 mmol scale with brominated monomer **4** and monomer **3** (1.2 equiv.). Column eluted with 1-4 % MeOH:DCM. Yield of 197 mg, 0.25 mmol (39 %) as a red solid. ^{1}H NMR

(400 MHz, $CDCl_3$): δ = 7.80 (2H, br s, -NHCOCO-), 5.07 (2H, br s, -NHBoc), 4.41-4.54 (8H, m, Ar-OCH_2-), 3.53-3.70 (20H, m, -OCH_2-), 3.34 (4H, dt, J_1 = J_2 = 5.1 Hz, -NHCH_2-), 1.44 (18H, s, Boc) ppm; ^{13}C NMR (100 MHz, $CDCl_3$): δ = 175.82 (-COCONHR), 161.68 (-COCONHR), 155.99 (-CO_2^{t}Bu), 149.43 (ArC3), 139.26 (ArC4), 123.32 (Ar$C_α$), 109.16 (Ar$C_α$), 79.19 (-CMe_3), 70.40 (-OCH_2CH_2-), 70.36 (-OCH_2CH_2-), 70.21 (-OCH_2CH_2-), 69.36 (-OCH_2CH_2-), 65.49 (Ar-OCH_2-), 64.62 (Ar-OCH_2-), 40.36 (-NHCH_2CH_2-), 39.30 (-NHCH_2CH_2-), 28.42 (-CMe_3) ppm; IR ($υ_{max}$, solid): 3335, 2974, 2938, 2869, 1682, 1620, 1516, 1471, 1427, 1360, 1269, 1247, 1167, 1071 cm^{-1}; HRMS m/z (ESI+): Found: 909.2851 (M+Na), Calc.: 909.2874.

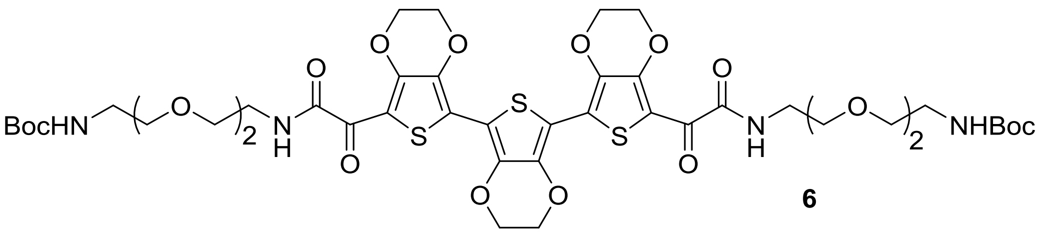

6

Run on 0.9 mmol scale with brominated monomer **4** and EDOT (0.55 equiv.). Column eluted with 1-6 % MeOH:DCM. Yield of 312 mg, 0.30 mmol (67 %) as a red solid. ^{1}H NMR (400 MHz, $CDCl_3$): δ = 7.82 (2H, br s, -NHCOCO-), 5.10 (2H, br s, -NHBoc), 4.41-4.53 (12H, m, Ar-OCH_2-), 3.52-3.66 (20H, m, -OCH_2-), 3.35 (4H, dt, J_1 = J_2 = 5.0 Hz, -NHCH_2-), 1.43 (18H, s, Boc) ppm; ^{13}C NMR (100 MHz, $CDCl_3$): δ = 175.03 (-COCONHR), 162.08 (-COCONHR), 156.99 (-CO_2^{t}Bu), 149.76 (ArC3), 140.14 (Ar$C_β$), 136.83 (Ar$C_β$), 125.23 (Ar$C_α$), 111.64 (Ar$C_α$), 109.13 (Ar$C_α$), 79.16 (-CMe_3), 70.38 (-OCH_2CH_2-), 70.35 (-OCH_2CH_2-), 70.22 (-OCH_2CH_2-), 69.42 (-OCH_2CH_2-), 65.49 (Ar-OCH_2-), 65.22 (Ar-OCH_2-), 64.53 (Ar-OCH_2-), 40.37 (-NHCH_2CH_2-), 39.26 (-NHCH_2CH_2-), 28.41 (-CMe_3) ppm; IR ($υ_{max}$, solid): 3344, 2974, 2932, 2870, 1703, 1678, 1604, 1518, 1470, 1427, 1360, 1268, 1247, 1167, 1070 cm^{-1}; HRMS m/z (ESI+): Found: 1027.3011 (M+H), Calc.: 1027.2987.

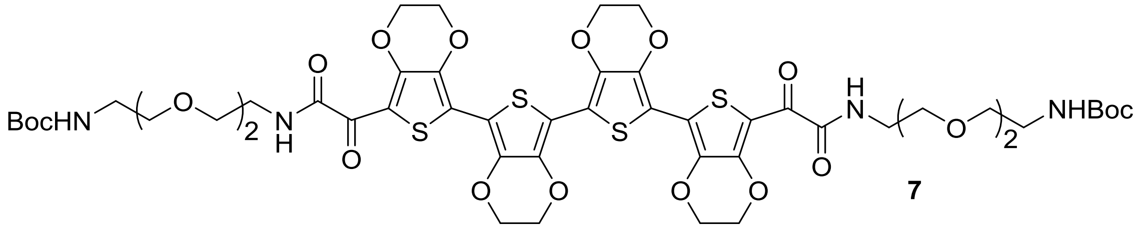

7

Run on 0.32 mmol scale with brominated dimer **18** and dimer **17** (1.2 equiv.). Column eluted with 1-4 % MeOH:DCM with 1 % NEt_3. Yield of 280 mg, 0.24 mmol (75 %) as a purple solid. Aromatic peaks could not be resolved in the 13C NMR spectra, even after long scan times at elevated temperatures. Details are therefore not given. ^{1}H NMR (400 MHz, $CDCl_3$): δ = 7.77 (2H, br s, -N$\underline{H}$COCO-), 5.01 (2H, br s, -N$\underline{H}$Boc), 4.27-4.45 (16H, m, Ar-OC$\underline{H}_2$-), 3.43-3.62 (20H, m, -OC$\underline{H}_2$-), 3.22-3.31 (4H, m, -NHC$\underline{H}_2$-), 1.37 (18H, s, $\underline{\text{Boc}}$) ppm; HRMS m/z (MALDI+): Found: 1166.2812 (M+H), Calc.: 1166.2834.

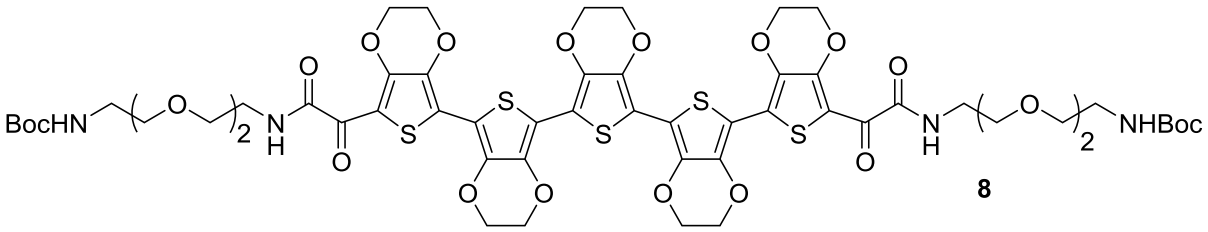

8

Run on 0.15 mmol scale with brominated dimer **18** and EDOT (0.5 equiv.). Column eluted with 1-4 % MeOH:DCM with 1 % NEt_3. Yield of 144 mg, 0.11 mmol (73 %) as a dark purple solid. Aromatic peaks could not be resolved in the 13C NMR spectra, even after long scan times at elevated temperatures. Details are therefore not given. ^{1}H NMR (400 MHz, $CDCl_3$): δ = 7.77 (2H, br s, -N$\underline{H}$COCO-), 5.01 (2H, br s, -N$\underline{H}$Boc), 4.29-4.47 (20H, m, Ar-OC$\underline{H}_2$-), 3.44-3.61 (20H, m, -OC$\underline{H}_2$-), 3.21-3.31 (4H, m, -NHC$\underline{H}_2$-), 1.37 (18H, s, $\underline{\text{Boc}}$) ppm; HRMS m/z (MALDI+): Found: 1306.2745 (M+H), Calc.: 1306.2766.

Hydrogelator synthesis

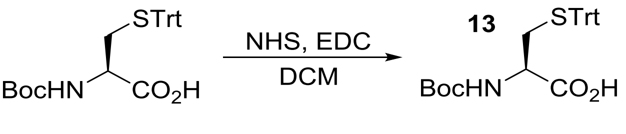

1-Ethyl-3-(3-dimethylaminopropyl)carbodiimide (618 mg, 3.2 mmol) was added to a solution of *N*-hydroxysuccinimide (273 mg, 2.4 mmol) and Boc-Cys(Trt)-OH (1 g, 2.2 mmol) in DCM (50 mL) and stirred for 2 hrs. The mixture was then washed with water (2 x 50 mL) and brine (150 mL), and the organics dried with $MgSO_4$, filtered, and concentrated *in vacuo* to provide the DP as a white solid. A yield of 1.2 g, 2.1 mmol (97 %) was obtained. Spectroscopic data were consistent with those previously reported.[3] ^{1}H NMR (400 MHz, $CDCl_3$): δ = 7.46 (6H, d, *J* = 7.5 Hz, ArH), 7.32 (6H, dd, $J_1 = J_2$ = 7.5 Hz, ArH), 7.25 (3H, t, *J* = 7.5 Hz, ArH), 4.89 (1H, br d, *J* = 7.7 Hz, -NH), 4.31-4.40 (1H, m, H_α), 2.67-2.88 (6H, m, H_β & -OSu), 1.45 (9H, s, Boc) ppm;

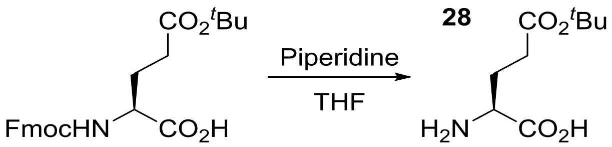

Fmoc-Glu(O^{t}Bu)-OH (2 g, 4.7 mmol) was added to a mixture of THF (40 mL) and piperidine (8 mL) and stirred for 1 h. The mixture was concentrated *in vacuo* and azeotroped with THF (2 x 50 mL). The residue was triturated for 1 h in diethyl ether (50 mL) and the resultant white solid collected by filtration, washed with diethyl ether (50 mL), and dried *in vacuo*. A yield of 0.9 g, 4.4 mmol (94 %) was obtained. Spectroscopic data were consistent with those previously reported.[4] ^{1}H NMR (400 MHz, MeOD): δ = 4.62 (2H, br s, -NH_2), 3.59 (1H, t, *J* = 12.0 Hz, H_α), 2.43-2.50 (2H, m, H_γ), 2.02-2.15 (2H, m, H_β), 1.47 (9H, s, -CO_2^{t}Bu) ppm;

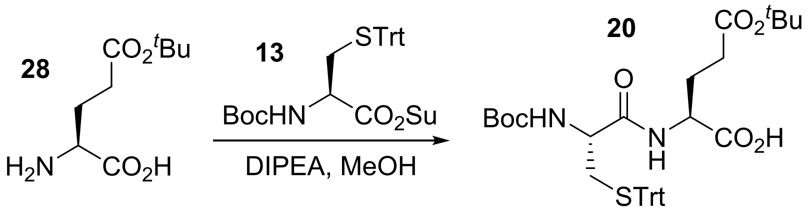

Boc-Cys(Trt)-OSu **13** (2 g, 3.6 mmol) was added to a solution of H_2N-Glu(O^{t}Bu)-OH **28** (800 mg, 3.9 mmol) and DIPEA (1.86 mL, 10.7 mmol) in methanol (50 mL). After stirring for 2 hrs the solvent was removed *in vacuo* and the residue redissolved in DCM (100 mL). The organics were washed with water (100 mL) and brine (100 mL), dried with $MgSO_4$, filtered,

and concentrated *in vacuo*. The residue was purified by flash column chromatography, eluting with 5-10 % MeOH:DCM. Pure fractions were concentrated *in vacuo* to give the DP as a colourless oil. A yield of 1.82 g, 2.8 mmol (79 %) was obtained. ^{1}H NMR (400 MHz, $CDCl_3$): δ = 7.42 (6H, d, *J* = 7.4 Hz, Trt), 7.29 (6H, dd, *J* = 7.4, 7.2 Hz, Trt), 7.22 (3H, t, *J* = 7.2 Hz, Trt), 7.01 (1H, br s, -NH), 4.96 (1H, br s, -NHBoc), 4.40 (1H, app br s, Glu-H_α), 3.92 (1H, app br s, Cys-H_α), 2.66-2.83 (1H, m, Cys-H_β), 2.45-2.60 (1H, m, Cys-H_β), 2.09-2.42 (3H, m, Glu-H_β & Glu-H_γ), 1.86-1.99 (1H, m, Glu-H_β), 1.35-1.46 (18H, m, Boc & -$CO_2{}^t$Bu) ppm; ^{13}C NMR (100 MHz, $CDCl_3$): δ = 174.4 (-CO_2R), 172.8 (-CO_2R), 170.47 (-CONHR), 155.34 (-NH$CO_2{}^t$Bu), 144.43 (Trt), 129.57 (Trt), 128.05 (Trt), 126.84 (Trt), 80.53 (-OCMe_3), 80.52 (-OCMe_3), 67.06 (Trt), 53.52 (Cys-C_α), 52.61 (Glu-C_α), 33.76 (Cys-C_β), 31.53 (Glu-C_γ), 28.28 (-CMe_3), 28.06 (-CMe_3), 27.46 (Glu-C_β) ppm; HRMS m/z (ESI+): Found: 671.2775 (M+Na), Calc.: 671.2761.

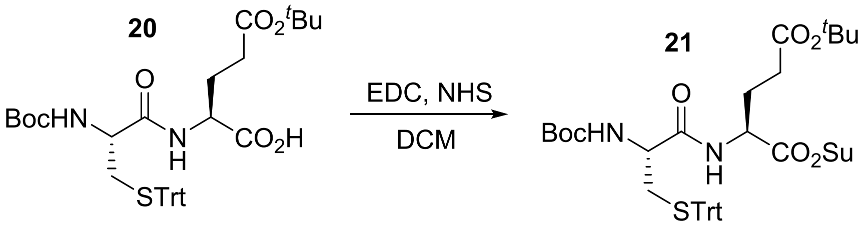

1-Ethyl-3-(3-dimethylaminopropyl)carbodiimide (345 mg, 1.8 mmol) was added to a solution of *N*-hydroxysuccinimide (156 mg, 1.35 mmol) and Boc-Cys(Trt)-Glu(O^{t}Bu)-OH **20** (800 mg, 1.2 mmol) in DCM (30 mL) and stirred for 2 hrs. The mixture was then washed with water (2 x 50 mL) and brine (150 mL), and the organics dried with $MgSO_4$, filtered, and concentrated *in vacuo* to provide the DP as a white solid. A yield of 1.2 g, 2.1 mmol (97 %) was obtained. The product was used directly in subsequent steps without further purification or analysis.

21 14

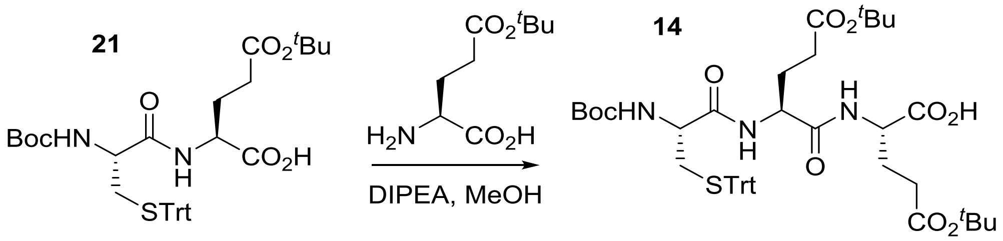

Boc-Cys(Trt)Glu(O^{t}Bu)-OSu **21** (2 g, 3.6 mmol) was added to a solution of H_2N-Glu(O^{t}Bu)-OH **28** (800 mg, 3.9 mmol) and DIPEA (1.86 mL, 10.7 mmol) in methanol (50 mL). After stirring for 2 hrs the solvent was removed *in vacuo* and the residue redissolved in DCM (100 mL). The organics were washed with water (100 mL) and brine (100 mL), dried with $MgSO_4$, filtered, and concentrated *in vacuo*. The residue was purified by flash column chromatography, eluting with 5-10 % MeOH:DCM. Pure fractions were concentrated *in vacuo* to give the DP as a colourless oil. A yield of 1.82 g, 2.8 mmol (79 %) was obtained. ^{13}C NMR was complicated by peak broadness and so analysis was undertaken. ^{1}H NMR (400 MHz, $CDCl_3$): δ = 7.42 (6H, d, *J* = 7.5 Hz, Trt), 7.29 (6H, dd, *J* = 7.5, 7.2 Hz, Trt), 7.17-7.25 (5H, m, Trt & -NH), 4.96 (1H, br s, -NHBoc), 4.28-4.54 (2H, m, Glu-H_α), 3.85-3.97 (1H, m, Cys-H_α), 2.47-2.86 (2H, m, Cys-H_β), 2.18-2.43 (4H, m, Glu-H_γ), 2.02-2.17 (2H, m, Glu-H_β), 1.83-2.01 (2H, m, Glu-H_β), 1.36-1.47 (27H, m, Boc & -$CO_2{}^t$Bu) ppm; HRMS m/z (ESI+): Found: 856.3839 (M+Na), Calc.: 856.3994.

Oligomer functionalisation

6 10

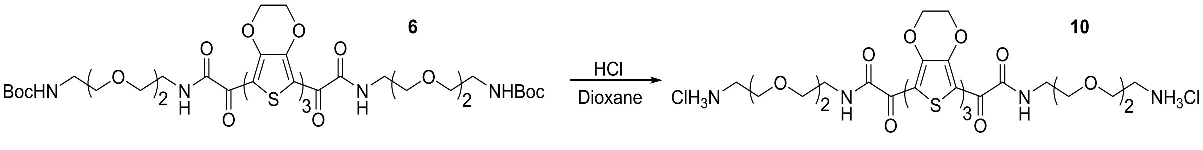

Hydrochloric acid (4 M in dioxane, 4 mL) was added to a solution of trimer **6** (350 mg, 0.34 mmol) in DCM (5 mL). After stirring for 2 hrs, the mixture was concentrated *in vacuo* and azeotroped with water (2 x 50 mL). The product was then dissolved in water (10 mL) and

lyophilised to give the DP as a red solid. A yield of 305 mg, 0.34 mmol (99 %) was obtained. The product was used in subsequent experiments without further characterisation.

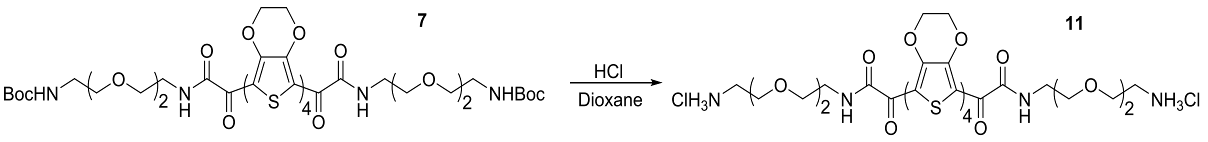

Tetramer **7** (250 mg, 0.21 mmol) was deprotected as described above. After concentrating *in vacuo* the product was azeotroped with DCM (3 x 20 mL) to give the DP as a purple solid. A yield of 218 mg, 0.21 mmol (99 %) was obtained. The product was used in subsequent experiments without further characterisation.

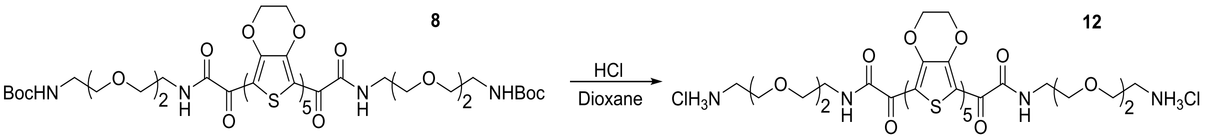

Pentamer **8** (180 mg, 0.14 mmol) was deprotected as described above. After concentrating *in vacuo* the product was azeotroped with DCM (3 x 20 mL) to give the DP as a dark purple solid. A yield of 165 mg, 0.14 mmol (99 %) was obtained. The product was used in subsequent experiments without further characterisation.

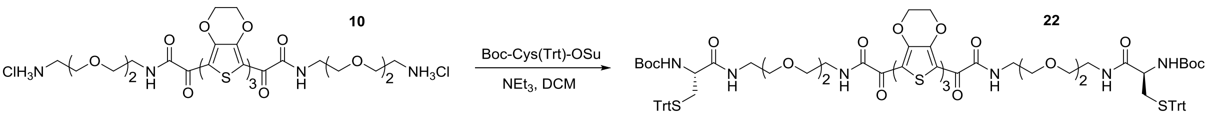

Triethylamine (25 μL, 180 μmol) was added to a suspension of trimer **10** (40 mg, 45 μmol) in DCM (3 mL), causing the oligomer to become dissolved. Boc-Cys(Trt)-OSu **13** (30 mg, 52 μmol) was then added and the mixture stirred for 18 hrs. The reaction was diluted with DCM (50 mL) and the organics washed with water (30 mL) and brine (30 mL), dried with $MgSO_4$, filtered, and concentrated *in vacuo*. The residue was purified by flash column chromatography, eluting with 3 % MeOH:DCM. Pure fractions were concentrated *in vacuo* to give the DP as a red oil. A yield of 77 mg, 44 μmol (98 %) was obtained.

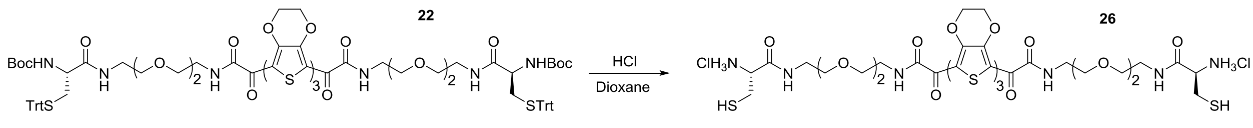

Hydrochloric acid (4 M in dioxane, 2 mL) was added to a solution of Cys-trimer **22** (77 mg, 45 µmol) in DCM (2 mL). After stirring for 3 hrs, the mixture was concentrated *in vacuo* and azeotroped with water (2 x 10 mL). The product was then dissolved in water (10 mL) and washed with diethyl ether (2 x 15 mL). The aqueous was lyophilised to give the DP as a red solid. A yield of 49 mg, 45 µmol (99 %) was obtained. HRMS m/z (MALDI+): Found: 1739.5204 (M+H), Calc.: 1739.5175.

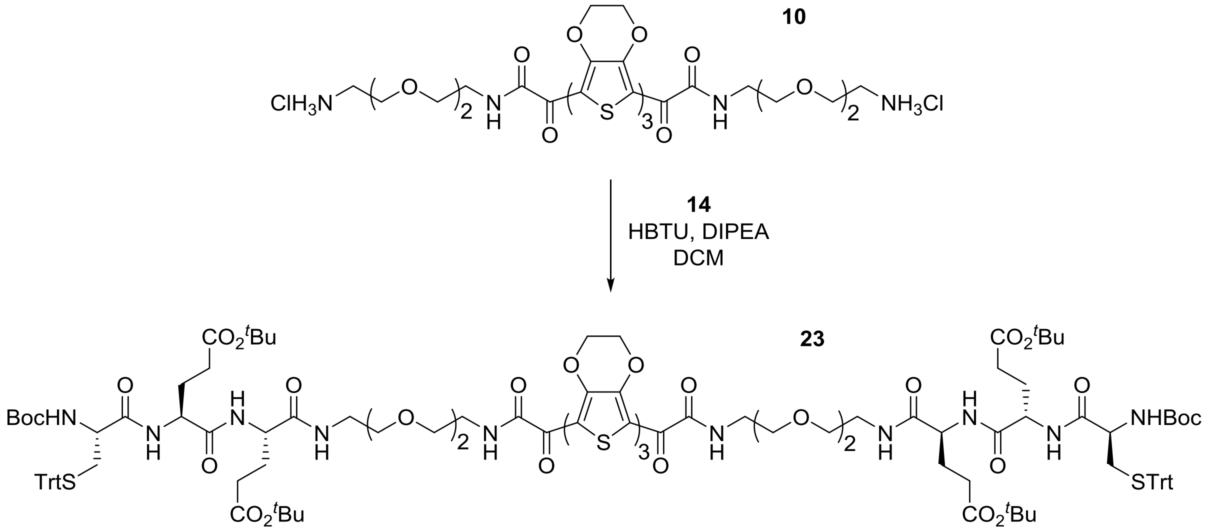

DIPEA (390 µL, 2.24 mol) was added to a suspension of trimer **10** (400 mg, 0.46 mmol) in DCM (30 mL), causing the oligomer to become dissolved. HBTU (424 mg, 1.12 mmol) and **14** (470 mg, 0.56 mmol) were then added and the mixture stirred for 18 hrs. The reaction was diluted with DCM (50 mL) and the organics washed with water (30 mL) and brine (30 mL), dried with $MgSO_4$, filtered, and concentrated *in vacuo*. The residue was purified by flash column chromatography, eluting with 1-4 % MeOH:DCM. Pure fractions were concentrated *in vacuo* to give the DP as a red oil. A yield of 320 mg, 0.13 mmol (46 %) was obtained.

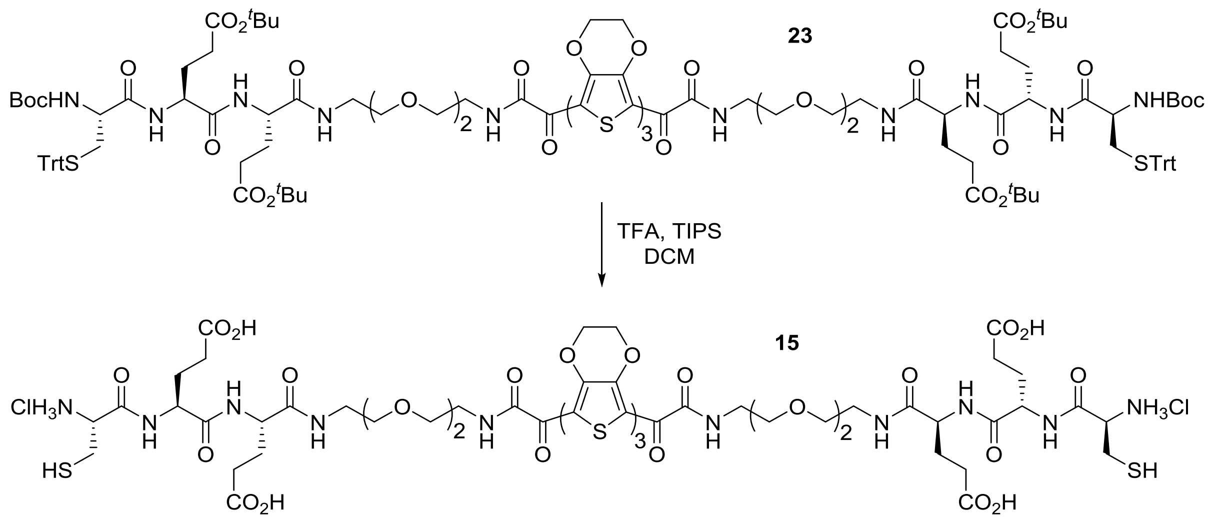

Trifluoroacetic acid (5 mL) was added to a solution of CysAspAsp-trimer **23** (320 mg, 130 μmol) and triisopropylsilane (160 μL, 780 μmol) in DCM (5 mL). After stirring for 3 hrs, the mixture was concentrated *in vacuo* and azeotroped with DCM (2 x 20 mL). The product was then dissolved in water (10 mL) and washed with diethyl ether (2 x 15 mL). The aqueous was lyophilised to give the DP as a red solid. A yield of 220 mg, 129 μmol (99 %) was obtained.

Amide-crosslinked hydrogel formation

Succinimide-PEG hub (Solution A)

8-arm carboxy-PEG (10 kDa, 50 mg, 5 μmol) was dissolved in DCM (2 mL). *N*-hydroxysuccinimide (5 mg, 45 mmol) and 1-Ethyl-3-(3-dimethylaminopropyl) carbodiimide hydrochloride (9.5 mg, 50 mmol) were then added and the reaction stirred for 18 hrs. The mixture was concentrated *in vacuo*, redissolved in DMSO (50 μL), and then diluted to a final volume of 500 μL with phosphate buffer (0.1 M, pH 7.8).

Amino-oligoEDOT crosslinker (Solution B)

Trimer **10** (5 mg, 6 μmol) was dissolved in water (150 μL).

Hydrogelation

Solution A (50 μL, 0.5 μmol hub) was added to Solution B (50 μL, 2 μmol crosslinker) and thoroughly mixed by pipetting up and down. During this time oligoEDOT crosslinker **10** was

buffered and quickly became poorly soluble. Solutions were left for 6 hrs to gel after which they were inverted and gently agitated. Although partial gelation had occurred, gels were highly heterogeneous and had weak mechanical properties that led them to partially disintegrate upon addition of water (1 mL).

Thiol-maleimide crosslinked hydrogel formation

Maleimide-PEG hub (Solution A)

8-arm maleimide-PEG (20 kDa, 5 mg, 0.2 μmol) was dissolved in phosphate buffer (50 μL, 0.2 M, pH 6).

Cys-oligoEDOT crosslinker (Solution B)

Trimer **26** (0.9 mg, 0.8 μmol) was poorly soluble in phosphate buffer and so was dissolved in water (50 μL).

CysAsp$_2$-oligoEDOT crosslinker (Solution C)

Trimer **15** (1.4 mg, 0.8 μmol) was dissolved in phosphate buffer (50 μL, 0.2 M, pH 6).

Hydrogelation

Solution A (10 μL, 40 nmol hub) was added to Solution B or C (10 μL, 160 nmol crosslinker) and rapidly pipetted to mix. Gelation occurred almost immediately and so the pipette was very quickly removed after mixing. Although gels could be formed using trimer **26**, they were highly heterogeneous due to precipitation of the oligomer upon buffering. Gels formed with trimer **15** were observed to have a far more homogeneous structure and could be easily handled within 15 min of addition. Gel size (10-200 μL) and polymer content (5-10 wt%) could be varied by scaling volumes and concentrations accordingly.

Tetramer and pentamer

Preparation of tetramer **26** and pentamer **27** stock solutions was attempted as described above. However, in both cases the oligomer was found to be insoluble in water and phosphate buffer. Attempts to pre-dissolve in DMSO followed by dilution in aqueous media were unsuccessful, with precipitation occurring immediately (Figure S5).

Oligomer leaching

Trimer **15** crosslinked PEG gels (25 μL, 5 wt %) were prepared as described above in a 96 well plate, varying the ratio of crosslinker to PEG hub (0.33-1.66 ratio thiol:maleimide). 10 minutes after gelation was initiated, 100 μL water was added and the plate agitated at room temperature. At set time points, 90 μL of supernatant was transferred to a fresh plate for absorbance measurements, and replaced with fresh water. Cumulative oligomer release was monitored over a period of 24 hrs, and compared to a standard concentration curve of oligomer **15** concentration to determine the quantities of release. Experiments were run in triplicate. It was found that a slight excess of thiol led to the most mechanically stable gels with decreased leakage (Figure S7).

Swelling

Trimer **15** crosslinked PEG gels (100 μL, 5 wt%) were prepared as described above. Gels were washed for 1 h in water (5 mL) and this process repeated 5 times to ensure removal of all soluble factors. Gels were lyophilised and weighed to measure their dry mass. Gels were then placed in buffer (0.2 M, 10 mL) at different pHs (citrate: pH 3; phosphate: pH 7; carbonate: pH 10). After swelling for 24 hrs, gels were removed, blotted gently to remove excess water, and reweighed. Swelling ratios were calculated by dividing swollen and dry masses. Experiments were run in triplicate (Figure S8).

PCL synthesis

Amine hydrochloride-capped oligomers (**10**, **11**, or **12**; 1 equiv) was dissolved in DCM and neutralised with trimethylamine (5 equiv.). The solvent was removed *in vacuo* and the residue

azeotroped with DCM (3 x 50 mL). ε-Caprolactone (210 equiv.), dried overnight over flame-dried 4 Å molecular sieves, and tin (II) octoate (0.01 equiv) were added under nitrogen and the mixture heated to 140 °C for 24 hrs. During this time the mixture became highly viscous. Polymers were partially cooled to 80 °C to prevent solidification, and precipitated in diethyl ether (100 mL). The resultant precipitate was collected by filtration, washed with diethyl ether (2 x 50 mL), and dried *in vacuo* to provide the desired PCL-oligoEDOT conjugates **16a-c** with a M_w ~ 25 kDa.

References

[1] D. Schumacher, J. Helma, F. A. Mann, G. Pichler, F. Natale, E. Krause, M. C. Cardoso, C. P. R. Hackenberger, H. Leonhardt; *Angew. Chem. Int. Ed.*, **2015**, *54*, 13787.

[2] S. J. Milner, A. Seve, A. M. Snelling, G. H. Thomas, K. G. Kerr, A. Routledge, A.-K. Duhme-Klair; *Org. Biomol. Chem.*, **2013**, *11* 3461.

[3] J. Kuil, M. J. E. Fischer, N. J. de Mol, R. M. J. Liskamp; *Org. Biomol. Chem.*, **2011**, *9*, 820-833.

[4] P. Betschmann, S. Sahli, F. Diedrich; *Helv. Chim. Acta*, **2002**, *85*, 1210.
